# Joint modelling of PSA dynamics and prostate cancer risks: A population-based study in men without prior prostate cancer

**DOI:** 10.1371/journal.pone.0343751

**Published:** 2026-07-10

**Authors:** Birzhan Akynkozhayev, Benjamin Christoffersen, Anna Lantz, Tobias Nordström, Keith Humphreys, Mark Clements

**Affiliations:** 1 Department of Medical Epidemiology and Biostatistics, Karolinska Institutet, Stockholm, Sweden; 2 Department of Urology, Karolinska University Hospital Solna, Stockholm, Sweden; 3 Department of Clinical Sciences, Danderyds Sjukhus, Stockholm, Sweden; Jan Biziel University Hospital No 2 in Bydgoszcz: Szpital Uniwersytecki Nr 2 im dr Jana Biziela w Bydgoszczy, POLAND

## Abstract

While the prostate-specific antigen (PSA) test is a widely used prostate cancer screening tool, its application remains controversial. Opportunistic PSA testing generates complex data in which testing intensities, PSA levels, and prostate cancer diagnosis are interdependent. Conventional analyses rarely model these processes jointly. The objective of this study was to develop a population-based joint model to analyse PSA dynamics, retesting patterns, and prostate cancer risk. We used the Stockholm Prostate Cancer Diagnostics Register to identify 506,761 men with at least one PSA test between 2003 and 2020. We fitted a joint model linking three components: a linear mixed-effects submodel for PSA over age, and two proportional hazards submodels for time to next PSA test and time to prostate cancer diagnosis. PSA increased nonlinearly with age, with substantial between-person heterogeneity and increasing unexplained variation with increasing age. Estimates from models fitted to each time-to-event process separately were attenuated relative to the joint model: hazard ratios per doubling of PSA were 1.61 (95% CI: 1.59-1.62; P < .001) versus 2.01 (95% CI: 1.99-2.02; P < .001) for prostate cancer diagnosis, respectively, and 1.068 (95% CI: 1.066-1.070; P < .001) versus 1.163 (95% CI: 1.161-1.165; P < .001) for retesting, respectively. This pattern is consistent with informative observation bias: men with higher PSA values are tested more frequently, so that part of the association between PSA and the event is absorbed by the unmodelled observation mechanism when the processes are analysed separately. Jointly modelling PSA, testing intensity, and diagnosis through shared random effects accounts for this dependence. As a limitation, the study is primarily limited by its observational nature and a lack of data on non-cancer factors that can elevate PSA, such as urinary tract infections or lower urinary tract symptoms. In conclusion, jointly modelling PSA dynamics and testing behaviour corrects for the informative observation bias inherent in opportunistic testing. This approach yields more accurate population estimates compared to traditional isolated models. Our findings suggest that PSA dynamics may be clinically informative and that screening models should jointly incorporate testing history and PSA trajectories to improve precision.

## Introduction

Prostate cancer is the most common cancer and is the third leading cause of cancer-related death among men in the European Union [1]. PSA is an inexpensive blood test that has long guided decisions on prostate cancer screening. The use of a single PSA threshold remains controversial [2–4]. The European Randomized Study of Screening for Prostate Cancer [5] and the Cluster Randomized Trial of PSA Testing for Prostate Cancer [6] demonstrated significant reductions in prostate cancer mortality, whereas the U.S. Prostate, Lung, Colorectal and Ovarian trial found no such reduction [7]. PSA levels increase with age and age-specific thresholds have been proposed to improve screening accuracy, yet their use remains controversial [8–10].

Longitudinal PSA trajectories may provide information beyond a single measurement. PSA doubling times have been linked to aggressive disease [11,12], although population-level studies have questioned their predictive value [13,14].

Large trials and health-system cohorts have documented extensive repeat PSA testing in routine care [15–20], but most analyses have considered only one of three key processes: longitudinal PSA trajectories, testing patterns (the observational process), or time to prostate cancer diagnosis. Few studies have applied joint modelling frameworks, which link longitudinal measurements with time-to-event outcomes, to PSA data, but these have focused on men after a prostate cancer diagnosis [21–23]. There is limited evidence on how PSA trajectories, repeat testing, and diagnosis are inter-related in men preceding any diagnosis of prostate cancer. Moreover, existing studies have not explicitly accounted for the fact that men with higher PSA values are more likely to undergo frequent testing. Recent computational developments now make a joint analysis of all three processes feasible for larger register-based datasets.

In this study, we develop a detailed population-based PSA model for men without a prior prostate cancer diagnosis. Using extensive PSA test data linked with rich health and population registers, we describe: (a) PSA trajectories, which are allowed to vary between individuals; (b) time to prostate cancer diagnosis; and (c) PSA testing patterns, with both diagnosis and testing dependent on the underlying PSA trajectory. Jointly modelling these longitudinal and time-to-event processes may provide novel insights into both the PSA dynamics and the rescreening behaviour. Specifically, we expect that estimates from the joint model will more accurately capture the underlying data-generating mechanisms than those from separate models. Patient-level posterior estimates of the random effects from such a model could serve as a basis for identifying men whose PSA levels are rising faster than expected of their age, signalling the need for closer clinical follow-up.

## Materials and methods

### Materials

This study uses data from the Stockholm Prostate Cancer Diagnostics Register [24], a comprehensive regional database that links information from Swedish national and regional registers, including PSA test dates and results, biopsies, cancer diagnoses, cause of death, and population data for men residing in Stockholm County. These data reflect a healthcare system where organised prostate cancer screening has not been recommended by the Swedish Board of Health and Welfare. Men may instead discuss prostate cancer testing with their clinician through a process of shared decision-making. Diagnostic practice for men who chose to have a PSA test evolved during the study window. In the earlier years of the cohort (approximately 2003–2015), an elevated PSA was typically followed by a systematic transrectal ultrasound-guided biopsy. Pre-biopsy magnetic resonance imaging (MRI) in men with elevated PSA has been recommended by international guidelines since 2015, and this recommendation was incorporated into the Swedish national guidelines (Nationellt vårdprogram för prostatacancer) in 2020 [25]. The contemporary pathway in Stockholm is therefore PSA-MRI-biopsy in selected men with an elevated PSA. The specific age-dependent PSA thresholds for referral into a standardised care pathway (Standardiserat vårdförlopp) are ≥ 3.0 ng/mL for men under 70 years, ≥ 5.0 ng/mL for those aged 70–80, and ≥ 7.0 ng/mL for those over 80. In clinical practice, if an initial PSA result is between 3 and 10 ng/mL and the prostate palpation is benign, a second PSA test is recommended after approximately three weeks to account for intra-individual variation before proceeding to MRI. Further, the indications for biopsy are refined using PSA density; for example, a biopsy is recommended for PI-RADS 3 findings only if the PSA density is ≥ 0.10 ng/mL/cm^3^, or for PI-RADS 1–2 findings if the density is ≥ 0.20 ng/mL/cm^3^.

The data were accessed on 12 January 2023 under the ethical approval from the Regional Ethical Review Board in Stockholm (Dnr 2012/438–31/3), with a subsequent amendment (Dnr 2016/620–32). As this was a register-based study, the requirement for individual informed consent was waived by the ethics committee. All data were processed anonymously, and the authors had no access to information that could be used to identify individual participants during or after the study.

The study population comprises 506,761 men who underwent at least one PSA test between 1 January 2003 and 31 December 2020. Disease verification was register-based and relied on biopsy-confirmed diagnoses, with predominantly systematic biopsies before 2018 and a rapidly increasing use of pre-biopsy MRI thereafter. PSA values and testing patterns change drastically after diagnosis and treatment, and the post-diagnosis is not the focus of this study; we therefore censored follow-up at the date of prostate cancer diagnosis and excluded all tests taken after the diagnosis date.

### Statistical methods

In outline, we fitted a joint model that linked three processes: the longitudinal trajectory of log-transformed PSA over age, time to prostate cancer diagnosis, and time to PSA test. The longitudinal submodel used a linear mixed-effects model to describe individual log-transformed PSA values as a nonlinear function of attained age, represented using a natural cubic spline with three degrees of freedom. Patient-level random intercepts and slopes were included to allow individual log-transformed PSA trajectories to deviate from the population-average trend.

Time to prostate cancer diagnosis was modelled using a left-truncated proportional hazards model, with the hazard proportional to: (i) a baseline hazard specified as a function of attained age using a natural cubic spline with three degrees of freedom; and to (ii) the underlying log-transformed PSA trajectory defined in the longitudinal submodel.

Time to PSA testing was modelled using a recurrent-event proportional hazards model in a counting-process formulation, with the hazard proportional to: (i) a baseline hazard specified as a function of attained age using a natural cubic spline with three degrees of freedom; and to (ii) the underlying log-transformed PSA trajectory from the longitudinal submodel. This formulation accounts for the correlation among repeated tests within individuals.

Correlations between the three processes were thus modelled through the shared underlying longitudinal PSA trajectory, which was modelled explicitly as a function of attained age. Each submodel can therefore be viewed implicitly as a model of attained age. Additionally, from the fitted joint model, we were able to estimate patient-specific trajectories for all three processes.

More formally, let individual i=1,…,n have $m_{i}$ PSA tests during the follow-up, with j-th test ($j=\text{1},\ldots ,m_{i}$) taken at age $t_{ij}$. Denote the corresponding log PSA value by $Y_{ij}$. The longitudinal submodel was


$$\begin{array}{c} Y_{ij}=X_{m}\left(t_{ij}\right)\beta_{m}+Z_{m}\left(t_{ij}\right)b_{i}+\epsilon_{ij}, \end{array}$$

where $X_{m}$ was the fixed-effects design matrix containing an intercept and a natural cubic spline on age at test with two interior knots placed at the tertiles of the observed age-at-test distribution, with $X_{m}\left(t_{ij}\right)$ representing the row corresponding to individual i at his jth test, $\beta_{m}$ was the vector of fixed-effects parameters. $Z_{m}$ was the random-effects design matrix, containing an intercept and a random slope for age, and $b_{i}$ were the individual-specific random effects. We assumed $b_{i}\sim N\left(\mu ,\Sigma \right)$ with mean vector μ and covariance matrix

$\Sigma =\;[\begin{array}{cc} \overset{\text{2}}{\underset{\text{0}}{\sigma }} & \rho \sigma_{\text{0}}\sigma_{\text{1}}\\ \rho \sigma_{\text{0}}\sigma_{\text{1}} & \overset{\text{2}}{\underset{\text{1}}{\sigma }} \end{array}]$,

where $\sigma_{\text{0}}$and $\sigma_{\text{1}}$ were the standard deviations of the random intercept and random slope, respectively, and ρ was their correlation. $\epsilon_{ij}\sim N\left(\text{0},\sigma^{\text{2}}\right)$ was the independent measurement error. Disregarding the sharing of random effects with the survival components, this is a standard linear mixed-effects model.

The hazard functions at age $t_{ij}$ were defined as


$$\begin{array}{c} h_{k}\left(t_{ij}\right)=\text{exp}\left(X_{s_{k}}\left(t_{ij}\right)\beta_{s_{k}}+\alpha_{k}Z_{m}\left(t_{ij}\right)b_{i}\right), \end{array}$$

where k=1 corresponded to the hazard of diagnosis and k=2 corresponded to the intensity of the next PSA test. $X_{s_{k}}$ was the design matrix for the fixed effects, specified similarly to the longitudinal submodel to include an intercept and a natural cubic spline on age with two interior knots placed at the tertiles of the observed event and censoring times. $\beta_{s_{k}}$ was the vector of fixed‐effects parameters, and $\alpha_{k}$ represented the association parameter linking the longitudinal random effects to each hazard process. The models accounted for left-truncation to adjust for the delayed entry of individuals at varying ages. Disregarding the sharing of random effects through $\alpha_{k}$, these are standard proportional hazards models with log-normal frailties.

The model was fitted using a frequentist marginal maximum likelihood approach. To approximate the intractable integrals over the random effects, we used Gaussian variational approximations [26]. Technical details of our novel implementation are provided in a separate paper [27]. To illustrate the fitted joint model, we presented three kinds of output: (i) the population-average log PSA trajectory as a function of age, derived from the fixed-effects part of the longitudinal submodel; (ii) hazard ratios per doubling of PSA for the diagnosis and retesting processes, obtained from the association parameters; and (iii) patient-specific estimated curves for all three processes, including the log PSA trajectory, the hazard of retesting, and the hazard of diagnosis, where each individual’s random effects were estimated from their variational posterior (analogous to best linear unbiased predictors in linear mixed-effects models) and combined with the corresponding population-average curve. For (iii), the uncertainty bands were derived from the variational covariance of the random effects.To contrast the joint model with modelling each process in isolation, we fitted corresponding standalone models to each component. Longitudinal PSA values were modelled using a linear mixed-effects model with attained age represented by a natural cubic spline with three degrees of freedom, matching the longitudinal submodel of the joint model. Time to prostate cancer diagnosis was modelled using a left-truncated proportional hazards model with attained age as the timescale, so the baseline hazard is a function of age, estimated non-parametrically. We additionally fitted a Royston-Parmar flexible parametric proportional hazards model, with the baseline log-cumulative hazard given by a natural cubic spline on age with three degrees of freedom, matching the joint model’s parameterisation. The PSA-testing process was modelled using a Prentice–Williams–Peterson (PWP) recurrent-event proportional hazards model in start–stop form, with the baseline stratified by event number and including a subject-specific frailty term to handle within-individual correlation among repeated tests; in the joint model, the same correlation is handled through the shared random effects.

All analyses were performed using R version 4.4.3, with the joint model fitted using the VAJointSurv package, and the standalone Cox and Royston-Parmar flexible parametric models fitted using the survival and rstpm2 packages, respectively.

## Results

Among the 506,761 men in the cohort, the median follow-up was 9.8 years, with a total of 1,836,900 PSA tests recorded before censoring at diagnosis (335,172 additional tests were taken after a prostate cancer diagnosis and were excluded). The median number of tests per man was two, and 303,752 men had at least two tests. After censoring at diagnosis, the median age at the first PSA test was 55.8 years. PSA values ranged from near zero to 22,510 ng/mL, with a median value of 1.2 ng/mL (IQR, 0.7–2.6 ng/mL). Overall, 82% of men with repeated PSA testing never reached the age-specific Swedish referral thresholds (≥3 ng/mL for men younger than 70 years, ≥ 5 ng/mL for those aged 70–80 years, and ≥7 ng/mL for those older than 80 years). During follow-up, 30,199 men (6.0%) were diagnosed with prostate cancer; the median age at diagnosis was 69.3 years (IQR, 63.1–75.7), and the median time from the first PSA test to diagnosis was 4.5 years (IQR, 0.0–9.5).

Table 1 provides an overview of the PSA testing patterns. Total PSA values together with rates for PSA testing, biopsies and diagnosis are higher at increasing ages.

**Table 1:** Summary of PSA testing, biopsy, and prostate cancer diagnosis by age group and calendar period, Stockholm 2003-2021.

Period	Age group (years)	Person-years (PY) (x1,000)	Tests total (x1,000)	Tests per PY	PSA, Median (IQR), ng/mL	Biopsies total	Biopsies per PY	Diagnosed total	Diagnosed per PY
2003–2008	<50	268.39	96.96	0.36	0.70 (0.50-1.00)	1,802	0.007	1,254	0.005
	50–59	269.34	174.01	0.65	0.90 (0.60-1.60)	7,966	0.030	6,743	0.025
	60–69	265.72	190.76	0.72	1.40 (0.80-2.80)	10,726	0.040	8,744	0.033
	70–79	152.73	113.46	0.74	2.50 (1.20-5.00)	6,109	0.040	4,646	0.030
	80+	88.39	61.51	0.70	3.40 (1.40-7.90)	2,086	0.024	1,694	0.019
2009–2014	<50	585.01	121.05	0.21	0.73 (0.50-1.10)	1,176	0.002	635	0.001
	50–59	733.51	215.78	0.29	0.97 (0.60-1.60)	6,696	0.009	4,094	0.006
	60–69	625.08	259.24	0.41	1.50 (0.81-2.80)	13,740	0.022	7,858	0.013
	70–79	274.34	140.31	0.51	2.20 (1.10-4.30)	7,950	0.029	4,163	0.015
	80+	133.12	66.33	0.50	3.30 (1.43-7.20)	2,752	0.021	1,490	0.011
2015–2021	<50	840.84	157.57	0.19	0.69 (0.47-1.00)	504	0.001	222	0.000
	50–59	799.40	234.64	0.29	0.94 (0.58-1.60)	3,551	0.004	1,529	0.002
	60–69	553.09	241.05	0.44	1.50 (0.80-2.80)	8,509	0.015	3,238	0.006
	70–79	405.96	198.72	0.49	2.00 (1.00-3.70)	9,010	0.022	3,158	0.008
	80+	154.61	72.28	0.47	2.70 (1.20-6.00)	3,198	0.021	1,046	0.007

### Joint model

**Fig 1:**
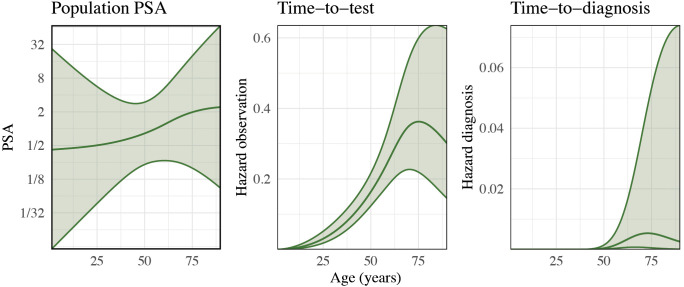
Estimated population-level longitudinal and time-to-event processes from the joint model.

Fig 1 displays the population-level estimates with age as the time scale. Looking at the three panels, we can see the three processes that form the core of the joint model: the longitudinal evolution of PSA (left), the age-specific hazard of undergoing a PSA test (middle), and the age-specific hazard of prostate cancer diagnosis (right). The green curves show population-level estimates with 95% pointwise prediction intervals that account for both fixed-effect uncertainty and between-individual variability.

### Longitudinal submodel

**Fig 2:**
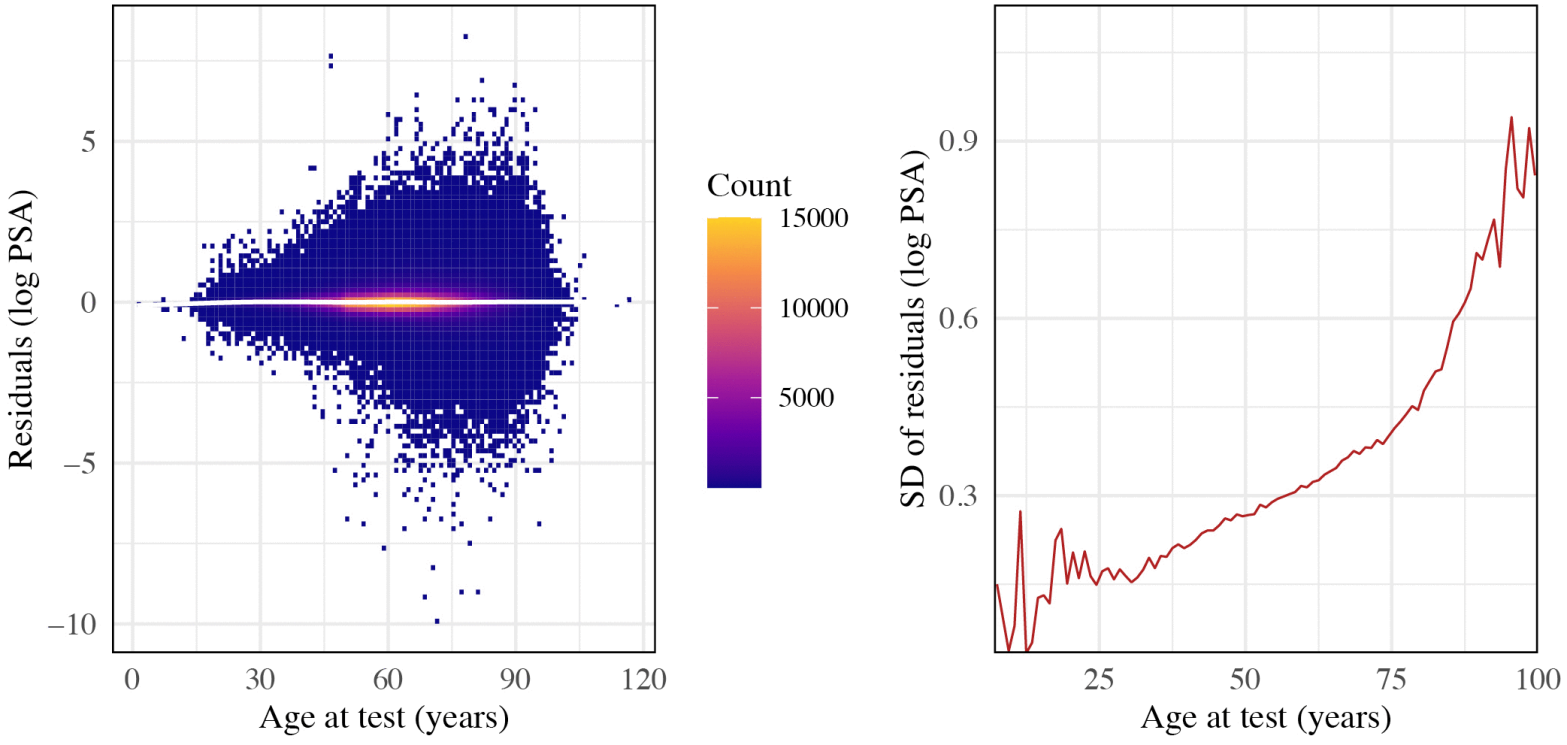
Heatmap (left) and standard deviations (right) of residuals from the longitudinal component of the joint model for log-transformed PSA by age at test.

The longitudinal submodel showed that population-average log PSA values increased nonlinearly with age. There was substantial heterogeneity in PSA trajectories across men, reflected by the large random-effect variances. The standard deviation of the random slope was 677.95 (95% CI: 674.74–681.17; P < .001), and the standard deviation of the random intercept was 0.812 (95% CI: 0.810–0.815; P < .001). The correlation between the intercept and slope was high at 0.59 (95% CI: 0.59–0.60; P < .001), indicating that men with higher baseline PSA tended to have steeper age-related increases. Additionally, we observed a steady increase in the standard deviation of the residuals (Fig 2), indicating heteroscedasticity. In other words, the magnitude of unexplained variation in log PSA grows larger with age.

These findings were consistent with the standalone longitudinal model (S1 File), although the joint model estimated a slightly lower population-average trajectory.

### Time-to-event submodels

A doubling of PSA concentration was associated with a higher hazard of diagnosis. This estimated effect was stronger than that obtained when modelling time to diagnosis separately; the Cox proportional hazards model specified using counting-processes formulation with log PSA as a time-updated covariate and robust standard errors clustered by individual, yielded an attenuated estimate. The Royston-Parmar flexible parametric model for time to diagnosis gave a hazard ratio per doubling of PSA of 1.61 (95% CI: 1.59–1.62; P < .001), identical to the Cox estimate.

For the observational process, the joint model indicated that doubling of PSA was positively associated with the hazard of a subsequent test. This was consistent with estimates from the PWP model fitted in isolation. Joint and standalone estimates for both processes are reported in Table 2.

**Table 2:** Hazard ratio per doubling of PSA from joint and standalone models for the diagnosis and retesting processes.

Process	Standalone model		Joint model	
	HR (95% CI)	P-value	HR (95% CI)	P-value
Time-to-diagnosis	1.61 (1.59-1.62)	<0.001	2.01 (1.99-2.02)	<0.001
Time-to-test	1.068 (1.066-1.07)	<0.001	1.163 (1.161-1.165)	<0.001

### Patient-specific estimated trajectories

**Fig 3:**
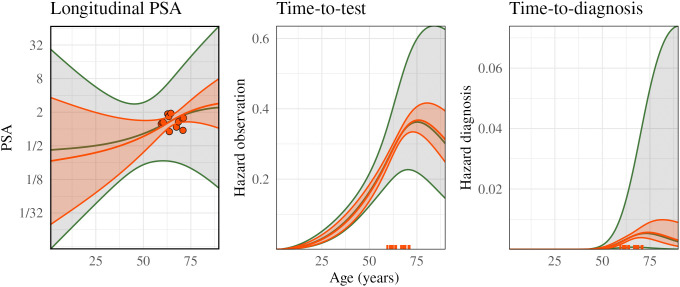
Estimated longitudinal and time-to-event processes from the joint model for an undiagnosed synthetic individual.

**Fig 4:**
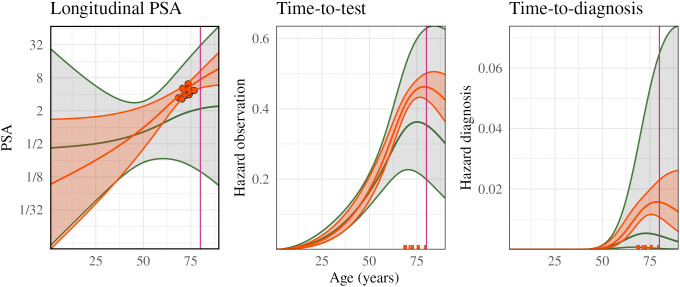
Estimated longitudinal and time-to-event processes from the joint model for a diagnosed synthetic individual; the vertical line represents the age at diagnosis.

Figs 3 and 4 illustrate fitted trajectories from the joint model for two individuals who were not diagnosed and diagnosed with prostate cancer, respectively. The green curves are population-level estimates from Fig 1. The orange curves represent individual-specific estimates based on the conditional modes of the random effects, with orange shaded areas representing 95% confidence intervals. For the diagnosed individual (Fig 4), the steadily rising PSA trajectory is accompanied by a sharp increase in both the hazard of subsequent testing and the hazard of diagnosis. In contrast, the undiagnosed individual (Fig 3) had PSA values that closely follow the population-average trajectory, with corresponding hazards for testing and diagnosis remaining consistent with population-average values. The individual-specific confidence intervals narrow across all three processes as more PSA measurements accumulate, most noticeably in the intervals where the tests are taken. It is important to note that these (orange) bands are within-sample posterior estimates conditional on the entire set of observed PSA values for that individual; they are not out-of-sample predictions and have not been validated as such (see Discussion for limitations on individual-level use). The individuals shown in Figs 3 and 4 are synthetic; the fitted curves were obtained from the joint model on the original data.

## Discussion

In this large, population-based study of more than half a million men in Stockholm County, we described how PSA values change with age, the observational process of retesting, and the time to prostate cancer diagnosis. Importantly, comparisons with isolated models demonstrated that standard approaches substantially underestimated the association between PSA and diagnosis by failing to account for the interdependence between biomarker trajectory, testing frequency, and cancer risk.

PSA values increased non-linearly with age and were highly heterogeneous across and within men. We observed considerable heteroscedasticity, with the variability in PSA values increasing with age. This pattern partially explains why using a single PSA threshold for screening in large populations is challenging.

Our comparison of the joint model and the linear mixed model for PSA trajectories highlights the importance of accounting for the observation process when analysing clinical biomarkers obtained through opportunistic or patient-driven testing. Men with elevated PSA levels are more likely to be re-tested, leading to an informative observation process in which the timing and frequency of PSA measurements depend on the underlying PSA trajectory. A mixed effects model assumes that measurement times are independent of the outcome, and consequently can lead to biased estimates. By jointly modelling the PSA process and the observation process, the joint model explicitly accounts for this dependency, therefore potentially providing more realistic estimates of population-level PSA trajectories. In our analysis, this adjustment resulted in slightly lower estimated mean PSA levels compared with the linear mixed-effects model, consistent with the expectation that failure to adjust for informative observation tends to overestimate PSA at the population level.

The mean age at diagnosis was 70.8 years, which is consistent with the higher PSA values observed among men aged 60 and older, likely reflecting cancers that had already developed by that age. However, clear differences in PSA distributions between diagnosed and undiagnosed men were also evident at younger ages.

When the longitudinal PSA process and the event processes were modelled separately, the estimated hazards for diagnosis and retesting were attenuated. For example, the hazard ratio for prostate cancer diagnosis per doubling of PSA was 1.61 in the separate Cox model but increased to 2.01 in the joint model. Similarly, the association between PSA and the risk of retesting rose from HR = 1.068 in the separate PWP model to HR = 1.163 under joint modelling. This attenuation in the separate analyses likely reflects informative observation bias: the timing of PSA measurements is not independent of the biomarker trajectory or the disease progression. When such feedback is ignored, part of the association between PSA and the outcome is absorbed into the unmodelled observation mechanism, leading to an underestimation of effect sizes. The joint model corrects for this by sharing random effects across the longitudinal and survival submodels, therefore accounting for the correlation between PSA levels, testing frequency, and diagnosis risk.

We used variational approximations to fit the joint model. This approach scaled well to a moderately large dataset and produced patient-level posterior estimates of the random effects as a by-product of fitting. These posterior estimates summarise how an individual deviates from the population-average PSA trajectory and come with measures of uncertainty derived from the variational parameters. They are, however, within-sample, conditional on each man’s full set of observed PSA values, and have not been developed or evaluated as a prediction tool in this study. They could form the basis of an individual-level prediction model in future work; such a model would additionally require a held-out validation sample not used in model fitting and an evaluation of calibration, discrimination, and clinical utility, none of which we report here. The patient-specific curves in Figs 3 and 4 should therefore be read as illustrations of the fitted random-effects structure rather than as validated predictions.

This study has several limitations. First, we do not explicitly model for a change of PSA trajectory after prostate cancer onset. Although cancer onset is not observable, inspection of individual PSA trajectories suggested that men who were subsequently diagnosed often experienced a more rapid rise in PSA after a certain point. To partially address this issue, we have censored from a prostate cancer diagnosis. Second, the data are observational, leading to substantial variation in the observation process across individuals. Our joint modelling approach seeks to explicitly model for this variation. Third, we lack information on other factors that can elevate PSA, such as urinary tract infections or lower urinary tract symptoms; the absence of these data may contribute to unexplained variability in PSA trajectories. Fourth, our findings may be specific to the Stockholm population. We posit that the connections between the three processes will be similar across populations. It would be useful to perform similar modelling in another population to test this hypothesis. A further limitation is that calendar time was not incorporated into the model. The estimates we report should therefore be interpreted as averaged over the 2003–2020 study period. As a sensitivity check, we refitted standalone time-to-event models within each of the three calendar periods used in Table 1. The hazard ratio per doubling of PSA was in the same direction and of similar magnitude in every period: for diagnosis, 1.71 (95% CI: 1.69–1.74; P < .001), 1.60 (95% CI: 1.58–1.62; P < .001), and 1.57 (95% CI: 1.54–1.59; P < .001); for retesting, 1.09 (95% CI: 1.09–1.09; P < .001), 1.08 (95% CI: 1.08–1.08; P < .001), and 1.05 (95% CI: 1.05–1.05; P < .001), in 2003–2008, 2009–2014, and 2015–2021, respectively. The modest attenuation across periods is consistent with changes in clinical practice during the study window, including the introduction of pre-biopsy MRI and changes in opportunistic testing intensity, but a clear PSA-event association is present in every period. Full details are given in S1 File.

Cancer onset is not observed in the data, but it has consequences for the PSA trajectory. When a cancer is present in the prostate, it leaks more PSA into the blood, so PSA levels in a man with undiagnosed cancer tend to rise faster than they would in a cancer-free man of the same age. The current joint model does not account for cancer onset. As a result, men who develop cancer during follow-up but have not yet been diagnosed contribute to the same average PSA trajectory as cancer-free men. Their faster PSA rise is absorbed by their individual random effects rather than being attributed to an underlying biological process.

Gulati et al. [28] addressed this by modelling log PSA as a piecewise linear function of age, with a single slope before cancer onset and a steeper slope after onset. Adapting this idea to our joint model would let us separate two effects that are currently mixed together: how PSA changes with age in cancer-free men, and how it changes after cancer develops. This extension is challenging because cancer onset times are not directly observed and enter the model as additional random effects to be inferred.

We did not account for clustering by general practitioner, clinic, or municipality. A multilevel analysis of PSA testing by municipality or health care provider would be interesting extensions.

In future work, we anticipate that this model can be used to estimate a detailed profile for PSA dynamics for use in a prostate cancer screening model, and estimate PSA uptake and rescreening to describe current opportunistic testing in a population. Additionally, we plan to first estimate the cancer onset distribution using a separate screening model, and then incorporate this estimated distribution into a changepoint model for the longitudinal PSA process.

## Conclusions

PSA trajectories, retesting behaviour, and prostate cancer diagnosis are strongly interdependent, and modelling them separately can substantially underestimate associations with PSA. Joint modelling accounts for informative observation in opportunistic testing and yields more accurate population-level estimates of all three processes. These findings support integrating PSA history and testing intensity into screening models to improve risk stratification and precision.

## Supporting information

S1 File Supplementary methods, figures, and tables. This file presents seasonal patterns in PSA testing volume (Fig S1); the longitudinal PSA process, including PSA probability densities by age group and by diagnosis status and a comparison of fixed-effect PSA trajectories from the joint and linear mixed models (Figs S2–S4); the observation (retesting) process (Fig S5); disease progression, including Kaplan–Meier estimates, Schoenfeld residuals for log PSA, and age-specific hazard ratios from a piecewise Cox model (Figs S6–S8 and Table S1); and a calendar-period sensitivity analysis of the standalone time-to-event models (Table S2).
(DOCX)
