# Supplementary material for: Joint modelling of PSA dynamics and prostate cancer risks: A population-based study in men without prior prostate cancer
Source: PLoS One. 2026 Jul 10;21(7):e0343751. doi: 10.1371/journal.pone.0343751 (PMC13354087; doi:10.1371/journal.pone.0343751)
Supplement: S1 File — This file presents seasonal patterns in PSA testing volume (Fig S1); the longitudinal PSA process, including PSA probability densities by age group and by diagnosis status and a comparison of fixed-effect PSA trajectories from the joint and linear mixed models (Figs S2–S4); the observation (retesting) process (Fig S5); disease progression, including Kaplan–Meier estimates, Schoenfeld residuals for log PSA, and age-specific hazard ratios from a piecewise Cox model (Figs S6–S8 and Table S1); and a calendar-period sensitivity analysis of the standalone time-to-event models (Table S2). (DOCX) [file pone.0343751.s001.docx]

# Supporting information

Please note that the main manuscript does not explicitly reference all supporting information.

## S1. PSA retesting seasonal patterns

PSA testing volumes from 2003 to 2020 are shown in Fig S1 and display a consistent seasonal pattern, with sharp declines during the summer months (July–August) and around the winter holidays (December–January). Testing peaks typically occurred in spring and autumn, reflecting the influence of healthcare system scheduling, holiday periods, and patient availability.


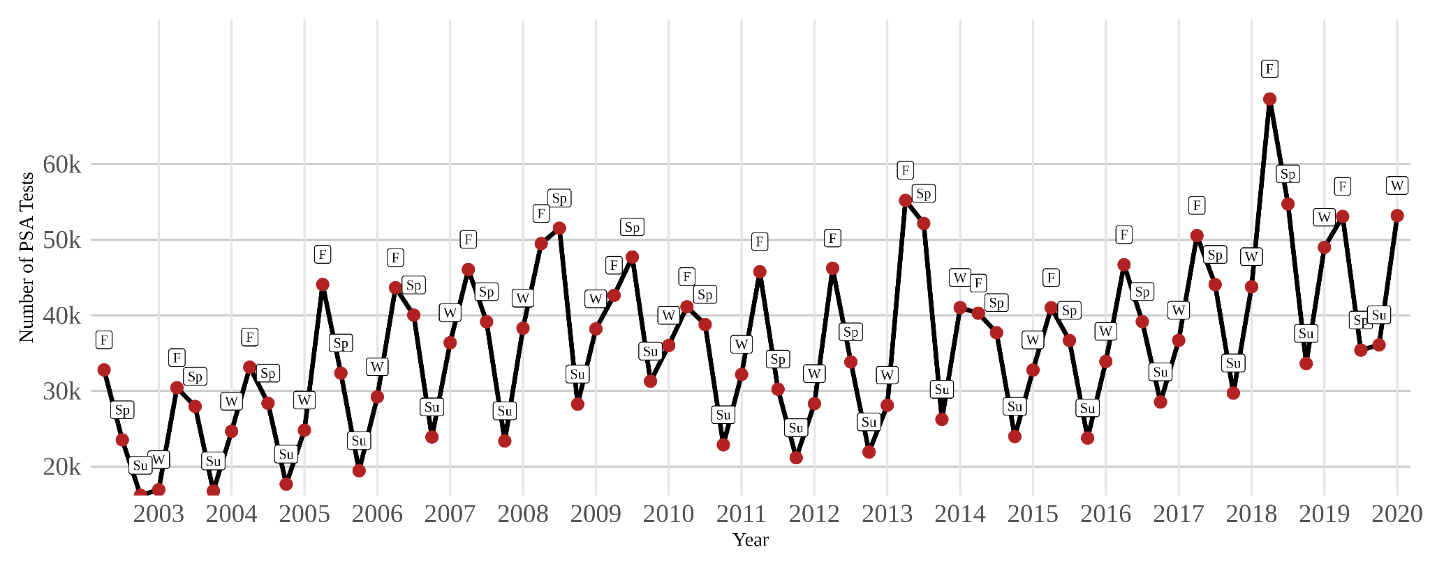


**Fig S1. Number of PSA tests by calendar season and year**

## S2. Longitudinal process for PSA values

PSA values increased with age, and their variance also increased with age; see Fig S2.

The Fig S2 displays that distributions of PSA values had long right tails across all age groups, indicating considerable heterogeneity and the presence of extreme PSA values. In addition, stratifying by diagnosis status showed that log-transformed PSA values were consistently higher among men who were eventually diagnosed with prostate cancer across all age groups (Fig S3).
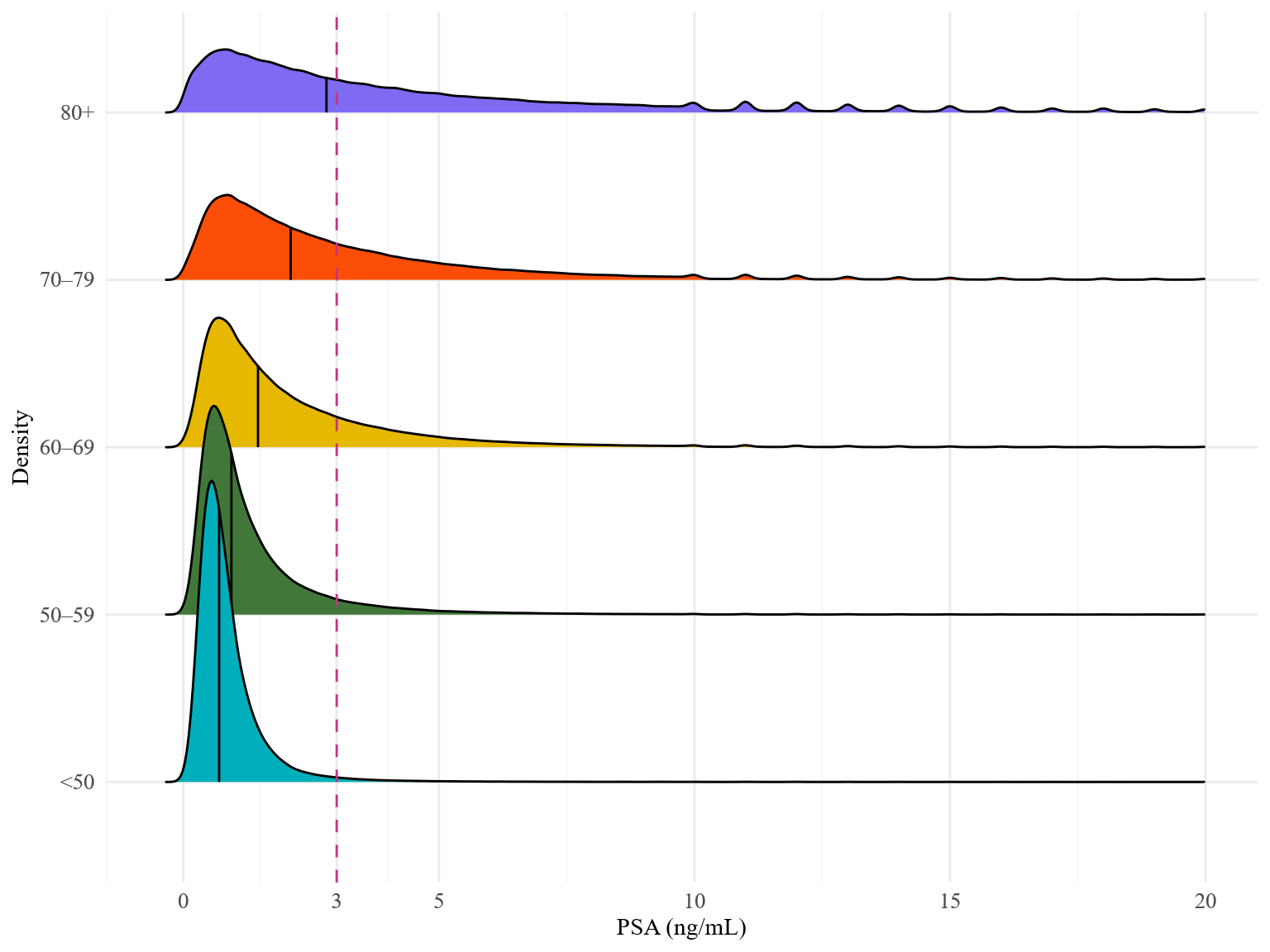


**Fig S2. PSA probability density by age group**, with vertical lines indicating the median values.


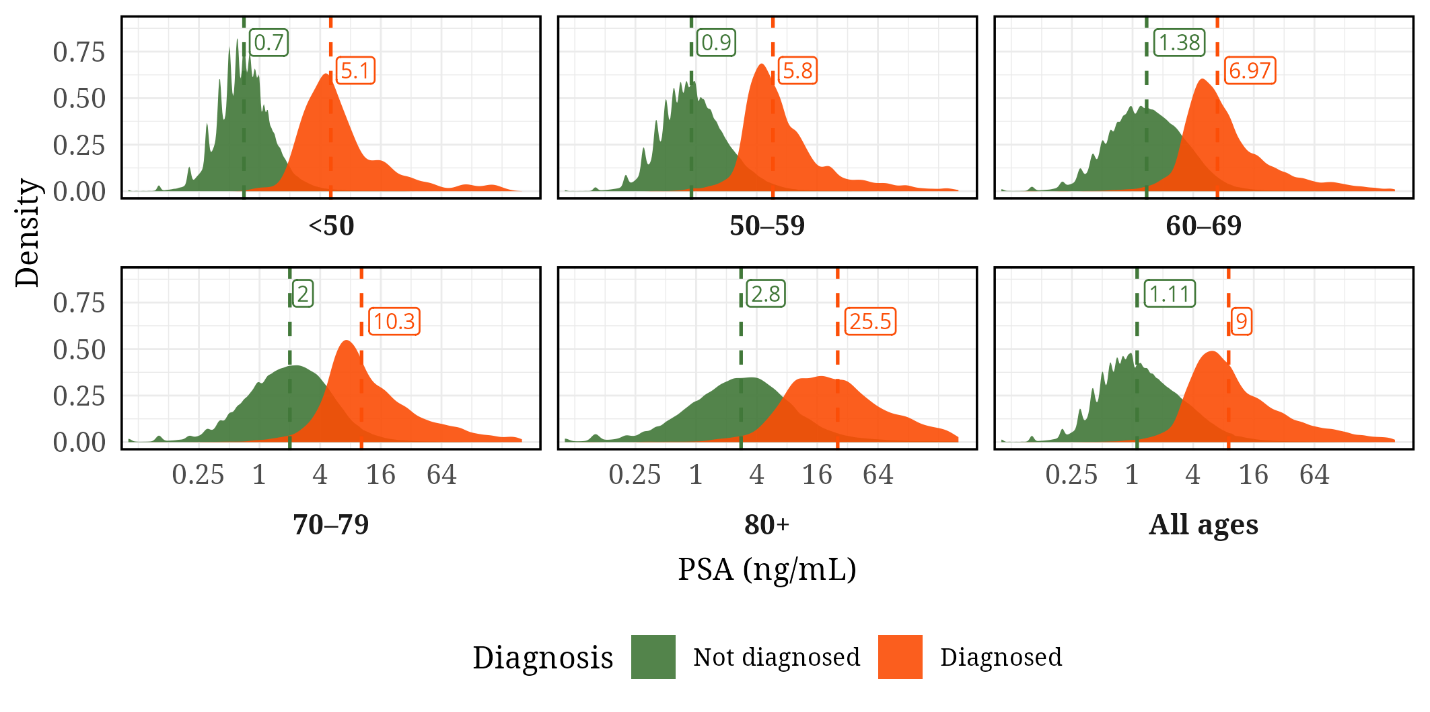
**Fig S3. Distribution of PSA by age group and diagnosis status (x-axis on a logarithmic scale).** For men diagnosed with prostate cancer, PSA measurements were restricted to tests obtained within 90 days prior to diagnosis. Vertical dashed lines indicate group-specific medians; box labels show the corresponding median PSA (ng/mL). Probability densities were estimated on the log PSA scale, but axis labels are displayed on the PSA scale for interpretability.

In a linear mixed-effects model of log-transformed PSA with age modelled by a natural cubic spline with three degrees of freedom, log PSA increased nonlinearly with age (all spline terms, <0.001). The fixed effects log PSA population mean is presented in Fig S4. The residual standard deviation was 0.42, showing considerable within-person variability.


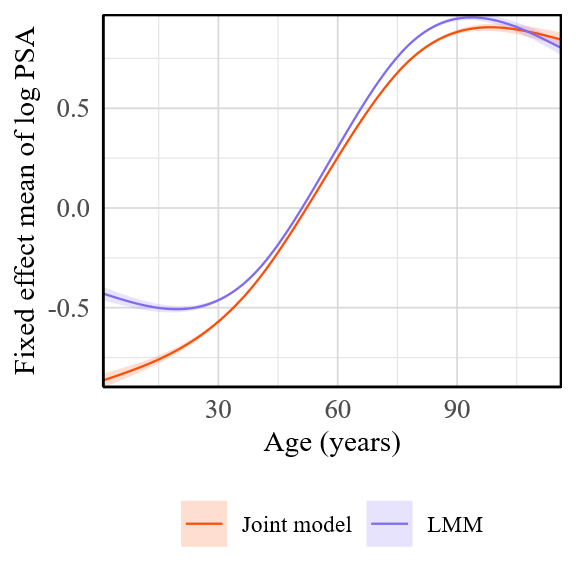


**Fig S4. Comparison of fixed-effect PSA trajectories from the joint model and the linear mixed model.**

## S3. Observation process

In the PWP gap-time model, doubling of PSA was associated with an increased hazard of subsequent testing (HR = 1.12; 95% CI = 1.12 to 1.12; p < 0.001).

Fig S5 represent the cumulative conditional probability of undergoing the next PSA test within 4 years since the previous test, given the individual has taken all preceding tests.


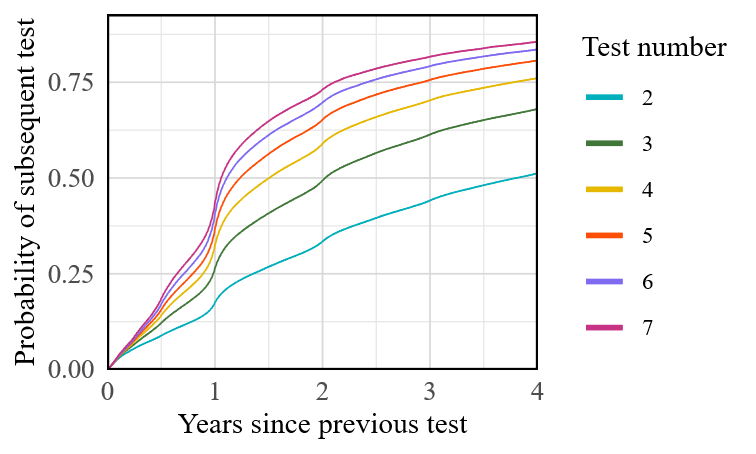


**Fig S5. Cumulative conditional probability of subsequent PSA testing by time since the previous test, stratified by test number.**

## S4. Disease progression

Left-truncated Kaplan–Meier curve for time to prostate cancer diagnosis, conditional on having at least one PSA test in the Stockholm region during 2002-2020 could be found in the Fig S6.


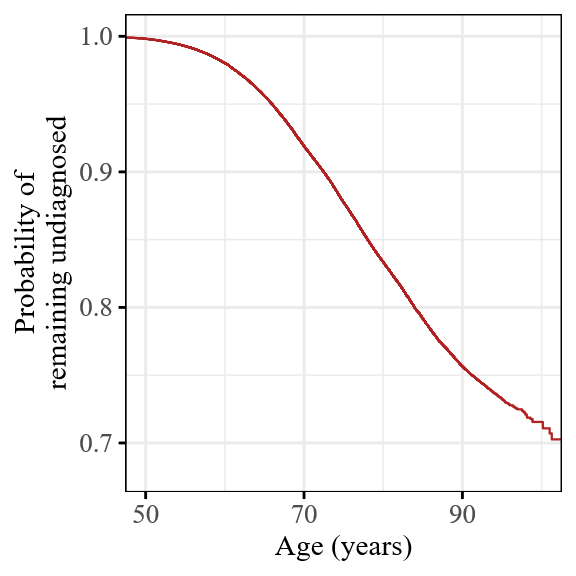


**Fig S6. Kaplan–Meier estimates of the probability of remaining undiagnosed.**

In the Cox proportional hazards model without time splitting we observed evidence of non-proportional hazards (Fig S7). The piecewise Cox model, which allowed the effect of log PSA to vary across age bands, is summarised in Fig S8. The full table of hazard ratios with 95% confidence intervals is provided in Table S1.


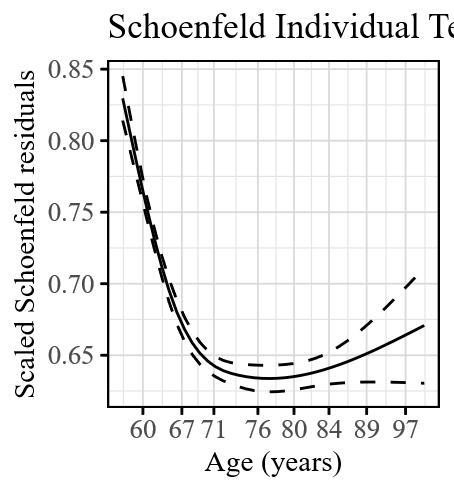


**Fig S7. Schoenfeld residuals and time-varying coefficient for log PSA from the Cox model for time to prostate cancer diagnosis.**


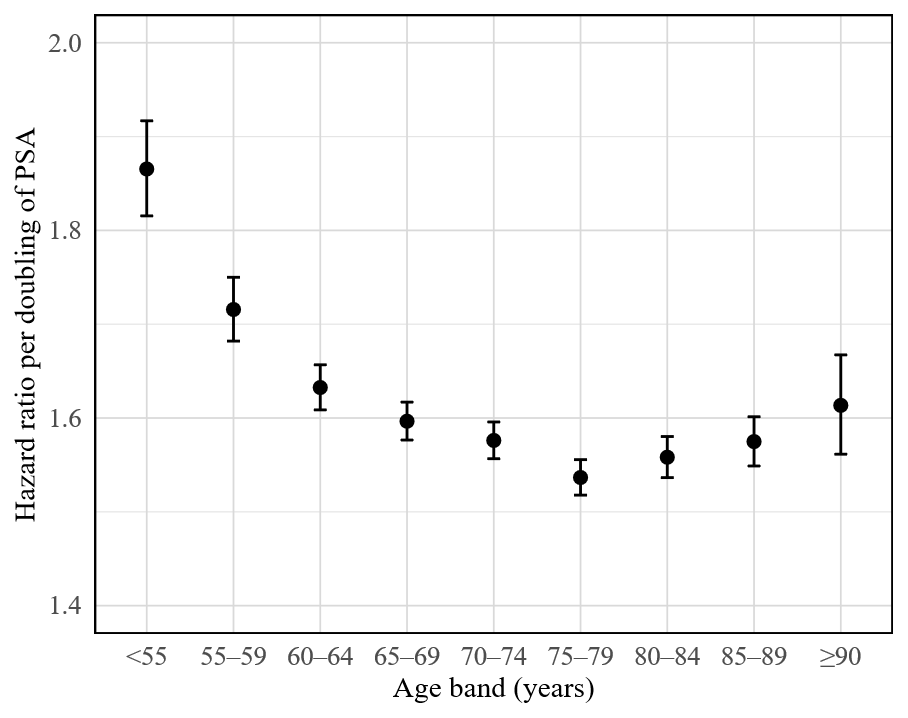


**Fig S8. Age-specific hazard ratios per doubling of PSA from a piecewise Cox model for time to prostate cancer diagnosis.**

| Age group | HR (95% CI) | P-value |
| --- | --- | --- |
| <55 | 1.865 (1.815-1.917) | <0.001 |
| 55–59 | 1.716 (1.682-1.750) | <0.001 |
| 60–64 | 1.633 (1.609-1.657) | <0.001 |
| 65–69 | 1.597 (1.577-1.617) | <0.001 |
| 70–74 | 1.576 (1.557-1.596) | <0.001 |
| 75–79 | 1.537 (1.518-1.556) | <0.001 |
| 80–84 | 1.558 (1.536-1.580) | <0.001 |
| 85–89 | 1.575 (1.549-1.601) | <0.001 |
| ≥90 | 1.613 (1.561-1.667) | <0.001 |

**Table S1. Age-specific hazard ratios per doubling of PSA from a piecewise Cox model for time to prostate cancer diagnosis.**

## S5. Calendar-period sensitivity analysis

The 2003–2020 study window spans a period during which opportunistic PSA testing intensity declined substantially in Stockholm and during which pre-biopsy MRI was introduced into the diagnostic pathway (around 2018, with formal incorporation into Swedish national guidelines in 2020). Calendar time was not modelled as a covariate in the joint model, primarily because adding a flexible calendar-time term to three submodels would have increased the parameter count and the runtime of an already expensive fit. As a sensitivity check, we refitted both standalone time-to-event models stratified by three calendar periods, matching the strata used in Table 1 of the main paper: 2003–2008, 2009–2014, and 2015–2021.

Each row in the analysis dataset was assigned to a calendar period based on the year of its PSA test date. A person who was tested in multiple periods therefore contributed data to each of those periods, reflecting the calendar time during which they were at risk of an event. The within-individual correlation across periods was accounted for through the cluster-robust standard errors at the person level. Across the three periods, the analysis dataset contained 636,696 rows in 2003–2008, 802,711 rows in 2009–2014, and 904,254 rows in 2015–2021.

**Time-to-diagnosis model:** the standalone Cox proportional hazards model, with attained age as the timescale, log-transformed PSA as a time-updated covariate, and cluster-robust standard errors at the person level, was refitted within each calendar period.

**Time-to-next-test model:** the standalone Prentice-Williams-Peterson recurrent-event model, in start–stop form with the baseline stratified by event number and cluster-robust standard errors at the person level, was refitted within each calendar period.

**Results:** estimates by calendar period are shown in Table S2.

| Process | Period | N events | HR (95% CI) | P-value |
| --- | --- | --- | --- | --- |
| Time to diagnosis | Pooled | 30,199 | 1.61 (1.59-1.62) | <0.001 |
|  | 2003-2008 | 10,725 | 1.71 (1.69-1.74) | <0.001 |
|  | 2009-2014 | 11,271 | 1.60 (1.58-1.62) | <0.001 |
|  | 2015-2021 | 8,203 | 1.57 (1.54-1.59) | <0.001 |
| Time to next PSA test | Pooled | 1,836,900 | 1.07 (1.07-1.07) | <0.001 |
|  | 2003-2008 | 541,844 | 1.09 (1.09-1.09) | <0.001 |
|  | 2009-2014 | 660,140 | 1.08 (1.08-1.08) | <0.001 |
|  | 2015-2021 | 634,916 | 1.05 (1.05-1.05) | <0.001 |

**Table S2. Hazard ratios per doubling of PSA by calendar period.**

# References

[1] Ormerod JT, Wand MP. Gaussian variational approximate inference for generalized linear mixed models. Journal of Computational and Graphical Statistics 2012;21. https://doi.org/10.1198/jcgs.2011.09118.
