# Supplementary material for: Joint modelling of PSA dynamics and prostate cancer risks: A population-based study in men without prior prostate cancer
Source: PLoS One. 2026 Jul 10;21(7):e0343751. doi: 10.1371/journal.pone.0343751 (PMC13354087; doi:10.1371/journal.pone.0343751)
Supplement: Attachment — Submitted filename: Response_to_Reviewers.pdf [file pone.0343751.s003.pdf]

# Response to Reviewers

We thank the reviewers for their careful reading of our manuscript. We believe that their comments have improved the paper, particularly the clarity of the methods and the framing of our findings. The reviewers' comments are copy-pasted, with our responses and the corresponding manuscript changes shown immediately below each point. Revised text from the manuscript is quoted in italics. All page and line numbers refer to the revised manuscript with tracked changes.

## Reviewer 1

Comments to the Author: The authors have analyzed PSA testing dynamics by applying a joint model of PSA values and testing patterns and risk of prostate cancer to observational data. The model identified stronger associations between PSA increase and prostate cancer diagnosis and retesting. They concluded that the joint approach is more appropriate compared to separate models and that PSA dynamics may be clinically informative.

The manuscript is well written and brings an important contribution to the field of modelling of PSA dynamics and screening. I have some (mainly minor) comments that may improve the manuscript.

**Response:** We appreciate the reviewer's suggestions on clarifying the target population, expanding the methodological detail, and adding an explicit comparison between the joint model and the separate models. We have sought to address each of these points in the revised manuscript. Individual responses follow below.

### Title

1. I suggest that you add the target population to the title – for example: "Joint modelling of PSA dynamics and prostate cancer risks: A population-based study of men without prior prostate cancer diagnosis."

   **Addressed:** We agree. Most of the existing joint-modelling literature on PSA concerns post-treatment follow-up, so making the pre-diagnosis target population explicit in the title is a useful clarification. We have adopted version of the reviewer's suggestion, the revised title now reads: *"Joint modelling of PSA dynamics and prostate cancer risks: A population-based study in men without prior prostate cancer."*

### Abstract

1. Without having read the rest of the manuscript, it is not entirely clear how the conclusions are supported by the results presented in the abstract alone. How does the joint model correct for bias, and in what sense is it more accurate? Could this be demonstrated with results, for example by explicitly comparing it with the separate models?

   **Addressed:** We have clarified these points in the abstract by: (i) quoting side-by-side estimates so the reader can see the magnitude of the attenuation in the models fitted in isolation; and (ii) stating the mechanism by which the joint model adjusts for an informative observation process. The revised abstract [page 2, line 28–36] now reads: *"Estimates from models fitted separately to each time-to-event process were attenuated relative to the joint model: the hazard ratios per doubling of PSA were 1.61 (95% CI: 1.59–1.62; $P < .001$) versus 2.01 (95% CI: 1.99–2.02; $P < .001$) for prostate cancer diagnosis, respectively, and 1.068 (95% CI: 1.066–1.070; $P < .001$) versus 1.163 (95% CI: 1.161–1.165; $P < .001$) for retesting, respectively. This pattern is consistent with an informative observation bias: men with higher PSA values are tested more frequently, so part of the association between PSA and the event is absorbed by the unmodelled observation mechanism when the processes are analysed separately. Jointly modelling PSA, testing intensity, and diagnosis through shared random effects accounts for this dependence."*

2. Minor: I do not agree that the following is a limitation "the model does not explicitly account for PSA trajectory changes after cancer onset" considering that you aimed to model PSA dynamics in men without prior prostate cancer diagnosis. It could perhaps be mentioned as a delimitation in the discussion.

**Addressed:** We have removed this statement from the abstract's limitations.

## Introduction

1. Minor: The last paragraph – I suggest that you add the target population to the aim, for example something like this: "In this study, we aimed to develop a detailed population-based PSA model for men without a prior prostate cancer diagnosis."

   **Addressed:** We agree and have updated the aim accordingly. The opening sentence of the final paragraph of the Introduction [page 5, line 71-72] now reads: *"In this study, we develop a detailed population-based PSA model for men without a prior prostate cancer diagnosis."*

## Materials and methods

1. The authors state that a PSA is followed by an MRI and then possibly by a biopsy. At the same time data on men that underwent PSA testing was collected between 2003-2020. MRI was only introduced into the diagnostic pathway during the latter part of the study window. I suggest that the authors clarify how the guidelines have changed over the study period and provide details on when MRI was introduced.

   **Addressed:** The reviewer is right that the original pathway description was written as if it applied throughout 2003–2020, when in fact diagnostic practice changed during the study window. We have rewritten the relevant paragraph in the Materials section to make the changes in diagnostic pathway explicit [page 5, line 90-95]: *"Diagnostic practice for men who had a PSA test evolved during the study window. In the earlier years of the cohort (approximately 2003 through 2015), an elevated PSA was typically followed by a systematic transrectal ultrasound-guided biopsy. Pre-biopsy magnetic resonance imaging (MRI) in men with elevated PSA has been recommended by international guidelines since 2015, and this recommendation was incorporated into the Swedish national guidelines (Nationellt vårdprogram för prostatacancer) in 2020 [25]. "*

2. Minor: Also, not all men should undergo an MRI before biopsy, so I suggest that you say that the contemporary typical diagnostic pathway is PSA-MRI-biopsy.

   **Addressed:** In addition to the previous comment we have also added pathway description [page 6, line 96-97]: *"The contemporary pathway in Stockholm is therefore PSA-MRI-biopsy in selected men with an elevated PSA."*

3. Minor: I would say that the paragraphs between lines 97 and 113 are results and should be moved to the results section.

   **Addressed:** We agree, and Reviewer 2 raised the same point. We have moved the descriptive content from the Materials section to the opening of the Results. The Materials section now retains only the cohort definition, disease verification, and censoring rules [page 7, lines 114–122]: *"The study population comprises 506,761 men residing in Stockholm County who underwent at least one PSA test between 1 January 2003 and 31 December 2020. Disease verification was register-based and relied on biopsy-confirmed diagnoses, with predominantly systematic biopsies before 2018 and a rapidly increasing use of pre-biopsy MRI thereafter. PSA values and testing patterns change drastically after diagnosis and treatment, and the post-diagnosis period is not the focus of this study; we therefore censored follow-up at the date of prostate cancer diagnosis and excluded all PSA tests taken after the diagnosis date."* The descriptive statistics on follow-up duration, number of tests, PSA distribution, referral-threshold crossings, and prostate cancer diagnoses now form a new opening paragraph of the Results section, immediately preceding Table 1 [page 11, lines 208–218].

4. Minor: what was the lower detection limit for PSA from the laboratories and did this vary over calendar time? Was there an upper reported limit for PSA? How did you handle this in the modelling?

   **Addressed:** We looked at this empirically. PSA values ranged from 0 to 22,510 ng/mL in the raw data, with no upper cap. The few zero values were replaced with the smallest non-zero value (0.0001 ng/mL) prior to the log transformation; all other values were used as reported. The minimum non-zero reported value varies over the study window, from 0.05 ng/mL in 2003–2005 to 0.0001 ng/mL in 2019–2020.

5. Given the emphasis on the novelty of the modelling approach, I would have appreciated a bit more detail about the model in the main text under "Statistical methods". For instance, how were the spline knots chosen, and how was the log transformed PSA trajectory explicitly incorporated into the subsequent Cox models?

**Addressed:** We agree with the reviewer that the main text needed more detail on the modelling specification. We have moved the formal mathematical description of the joint model from the Supporting information into the Statistical methods section [page 8, lines 153–177], so that both the spline knot placement and the explicit link between the longitudinal log PSA trajectory and the hazard submodels are visible in the main text. The revised text reads:

*"More formally, let individual $i = 1, \ldots, n$ have $m_i$ PSA tests during the follow-up, with the $j$-th test ($j = 1, \ldots, m_i$) taken at age $t_{ij}$. Denote the corresponding log PSA value by $Y_{ij}$. The longitudinal submodel was*

$$Y_{ij} = X_m(t_{ij})\beta_m + Z_m(t_{ij})b_i + \epsilon_{ij},$$

*where $X_m$ was the fixed-effects design matrix containing an intercept and a natural cubic spline on age at test with two interior knots placed at the tertiles of the observed age-at-test distribution, with $X_m(t_{ij})$ representing the row corresponding to individual $i$ at the $j$-th test; $\beta_m$ was the vector of fixed-effects parameters; and $Z_m$ was the random-effects design matrix, containing an intercept and a random slope for age, and $b_i$ were the individual-specific random effects. We assumed $b_i \sim N(\mu, \Sigma)$ with mean vector $\mu$ and covariance matrix $\Sigma$, and $\epsilon_{ij} \sim N(0, \sigma^2)$ was the independent measurement error. Disregarding the sharing of random effects with the survival components, this is a standard linear mixed-effects model.*

*The hazard functions at age $t_{ij}$ were defined as*

$$h_k(t_{ij}) = \exp(X_{s_k}(t_{ij})\beta_{s_k} + \alpha_k Z_m(t_{ij})b_i)\,,$$

*where $k = 1$ corresponded to the hazard of diagnosis and $k = 2$ corresponded to the intensity of the next PSA test. $X_{s_k}$ was the design matrix for the fixed effects, specified similarly to the longitudinal submodel to include an intercept and a natural cubic spline on age with two interior knots placed at the tertiles of the observed event and censoring times. $\beta_{s_k}$ was the vector of fixed-effects parameters, and $\alpha_k$ represented the association parameter linking the longitudinal random effects to each hazard process. The models accounted for left-truncation to adjust for the delayed entry of individuals at varying ages. Disregarding the sharing of random effects through $\alpha_k$, these are standard proportional hazards models with log-normal frailties.*

To clarify, the spline knots were placed at the tertiles of the relevant observed age distribution (the age-at-test distribution for the longitudinal submodel, and the event/censoring-time distribution for the hazard baselines), and the log PSA trajectory enters each hazard submodel through the random-effects design $Z_m(t_{ij})b_i$ scaled by the association parameter $\alpha_k$.

6. Minor: It would also have been good with a sentence or two about how the model was fitted, and a short description of how you plan to illustrate or present the fitted model(s). This would be valuable especially to those unfamiliar with the joint/latent modelling approach.

   **Addressed:** The model-fitting procedure is now described in the revised Statistical methods section [page 9, lines 175–180]: *"The model was fitted using a frequentist marginal maximum likelihood approach. To approximate the intractable integrals over the random effects, we used Gaussian variational approximations [26]. Technical details of our novel implementation are provided in a separate paper [27]."*

   and [page 11, line 203–206]: *"All analyses were performed using R version 4.4.3, with the joint model fitted using the VAJointSurv package, and the standalone Cox and Royston-Parmar flexible parametric models fitted using the survival and rstpm2 packages, respectively."*

   As suggested, we have additionally added a description of how the fitted joint model is presented in the Results, to help readers unfamiliar with joint modelling [page 10, lines 181–189]: *"To illustrate the fitted joint model, we presented three kinds of output: (i) the population-average log PSA trajectory as a function of age, derived from the fixed-effects part of the longitudinal submodel; (ii) hazard ratios per doubling of PSA for the diagnosis and retesting processes, obtained from the association parameters; and (iii) patient-specific estimated curves for all three processes, including the log PSA trajectory, the hazard of retesting, and the hazard of diagnosis, where each individual's random effects were estimated from their variational posterior (analogous to best linear unbiased predictors in linear mixed-effects models) and combined with the corresponding population-average curve. For (iii), the uncertainty bands were derived from the variational covariance of the random effects."*

7. Why did the separate models not include splines, or did they? It is not clear based on reading lines 135–139. I would have expected the separate models to be as similar as possible to the joint model in terms of how the covariates were represented.

**Addressed:** The reviewer is right that the methods text was unclear on this. The longitudinal submodel in the standalone analysis uses the same natural cubic spline (3 df) on attained age as the joint model. For the time-to-diagnosis model, the standalone Cox proportional hazards fit uses attained age as the timescale, so its baseline hazard is automatically a function of age, but estimated non-parametrically, whereas the joint model represents the same baseline parametrically as a natural cubic spline with 3 df. We additionally fitted the standalone diagnosis model as a Royston–Parmar flexible parametric proportional hazards model (rstpm2 package in R), with the baseline log-cumulative hazard given by a natural cubic spline on age with 3 df, matching the joint model. The hazard ratio per doubling of PSA was identical to the Cox estimate at this precision (1.61, 95% CI 1.59–1.62). We have retained the Cox model in the main text as our original choice, since it is the conventional first analysis for time-to-event data, and because it produces the same estimate as the spline-matched version; we have additionally added the Royston-Parmar flexible parametric model to the paper. For the PSA-testing process, the standalone PWP and the joint model use related but not identical mechanisms to handle within-person correlation among repeated tests. The standalone PWP stratifies the baseline by event number, uses the observed log PSA as a time-updated covariate, and includes a person-level frailty. The joint model uses a single parametric (natural cubic spline) baseline on age, links the testing hazard to the fitted random-effects component of the longitudinal log PSA trajectory, and uses random effects that are shared between the longitudinal and testing submodels. This is the closest feasible comparison: a flexible parametric recurrent-event model with a spline baseline and person-level random effects, which would more directly mirror the joint model's structure, did not converge in 2 days using 30 cores, so we did not wait for longer. The methods text has been clarified accordingly [page 10, lines 190–202]: *"To contrast the joint model with modelling each process in isolation, we fitted corresponding standalone models to each component. Longitudinal PSA values were modelled using a linear mixed-effects model with attained age represented by a natural cubic spline with three degrees of freedom, matching the longitudinal submodel of the joint model. Time to prostate cancer diagnosis was modelled using a left-truncated proportional hazards model with attained age as the timescale, so the baseline hazard is a function of age, estimated non-parametrically. We additionally fitted a Royston-Parmar flexible parametric proportional hazards model, with the baseline log-cumulative hazard given by a natural cubic spline on age with three degrees of freedom, matching the joint model's parameterisation. The PSA-testing process was modelled using a Prentice–Williams–Peterson (PWP) recurrent-event proportional hazards model in start–stop form, with the baseline stratified by event number and including a subject-specific frailty term to handle within-individual correlation among repeated tests; in the joint model, the same correlation is handled through the shared random effects."*

8. Minor: what was the covariance matrix for the random effects in the longitudinal submodel?

   **Addressed:** The covariance matrix of the random effects in the longitudinal submodel is an unstructured 2 by 2 matrix. We have added the covariance matrix structure to the Statistical methods section [page 9, lines 162–165]:

   *"...and covariance matrix*

$$\Sigma = \begin{pmatrix} \sigma_0^2 & \rho\,\sigma_0\sigma_1 \\ \rho\,\sigma_0\sigma_1 & \sigma_1^2 \end{pmatrix},$$

   *where $\sigma_0$ and $\sigma_1$ were the standard deviations of the random intercept and random slope, respectively, and $\rho$ was their correlation."*

   The estimated values of $\sigma_0$, $\sigma_1$, and $\rho$ are already reported in the Results section [page 14, lines 236–239].

9. Minor: supplement – I think there should be an explicit reference to the arXiv article that describes the method with the relevant identifier.

   **Addressed:** We now cite the paper containing technical details of the joint model implementation in the main manuscript. We have also updated the reference to include the explicit arXiv identifier, in PLOS/Vancouver format.

## Results

1. The results are clearly presented. However, I believe it would be valuable to include an explicit comparison between the joint model and the separate models in the manuscript, especially since this is a key finding highlighted in both the abstract and the discussion's summary of results. Perhaps Figures 1 and 3–4 could be combined to make space for additional details on all the models currently found in the supplement.

**Addressed:** We have added an explicit joint against standalone comparison to the main text as Table 2, complementing the additional methodological details on the standalone models added in response to Materials and methods section comment 7. The table summarises the hazard ratio per doubling of PSA from the standalone and joint models side-by-side for both the diagnosis and retesting processes. The relevant Results paragraph has been adjusted so that the four hazard ratios are not repeated [page 15, lines 248–260].

2. Minor: how come there is such a wide interval for PSA for younger ages in Figure 1? Is it for the same reason as for the later figures, or is there a larger heterogeneity in younger men?

   **Addressed:** The shaded band in Fig 1 is a 95% pointwise interval that incorporates between-individual variability through the random-effects component of the longitudinal trajectory ($\mathbf{Z}_m(t)\,\mathbf{b}_i$, where $\mathbf{b}_i$ is the individual's vector of random intercept and slope on age). The width of this band depends on a variance term that involves both random effects and the age $t$. Because the random intercept and random slope are correlated, the variance term is not constant across age: with our fitted covariance matrix, it is smallest somewhere in middle age and increases toward both the younger and older ends of the age range. The wider band at younger ages, and the (somewhat wider still) band at older ages, are both consequences of this.

   This is therefore a feature of the model's variance structure, not a sign that PSA values are more heterogeneous in younger men. The opposite is in fact true: Fig S2 of the Supporting information shows the empirical PSA density in five age groups, and the spread of PSA values is smallest in men under 50 and widens with each older age band. The same variance mechanism contributes to the wider intervals at the extremes of the age range in Figs 3 and 4, although those figures additionally reflect individual-level uncertainty about the random effects at ages outside an individual's own observed range.

3. Minor: did model performance somehow deteriorate or improve over calendar time, considering the changing diagnostic landscape and in particular the introduction of MRI?

   **Addressed:** This is closely related to Reviewer 2's comment 2 on calendar-time effects, and we have addressed it together with that comment. Full details are given in the revised Discussion [page 20, lines 357–368] and in Supporting information S5.

## Discussion / Conclusions

1. Minor: consider to give an example of how the model can be used in a personalized decision-making context?

   **Addressed:** We appreciate the suggestion. After careful consideration of Reviewer 2's comments, we have decided not to focus on individual-level predictions in this paper. Please see our response to Reviewer 2, comment 7, where we explain in detail how we have stepped back from a prediction-oriented framing throughout the manuscript.

2. I would have appreciated a more in-depth discussion of the modelling choices and the plausibility of the underlying assumptions. For example, the authors conclude the discussion by stating that they plan to model cancer onset and integrate it into the joint model. It would be helpful for readers to understand why this addition would be valuable – for example, in what ways might it improve model performance? Could the authors illustrate how the current model may underperform, perhaps by showing that it does not fully capture the heterogeneity and dynamics in the data over time, and how the proposed extension might address this limitation?

   **Addressed:** We agree that the rationale for the proposed cancer onset extension could be explained better. We have expanded the Discussion to explain what is currently missing in the model and why a cancer-onset extension would address it. The current joint model does not account for cancer-onset, so men who develop cancer during follow-up but have not yet been diagnosed contribute to the same average PSA trajectory as cancer-free men; their faster PSA rise is absorbed by their individual random effects rather than being attributed to an underlying biological process. We have linked the proposed extension to the prostate cancer natural history model of Gulati et al. (2010), who model log PSA as a piecewise linear function of age, with a single slope before onset and a steeper slope after onset. The revised text is at [page 20, lines 369–375]:

   *Cancer onset is not observed in the data, but it has consequences for the PSA trajectory. When a cancer is present in the prostate, it leaks more PSA into the blood, so PSA levels in a man with undiagnosed cancer tend to rise faster than they would in a cancer-free man of the same age. The current joint model does not account for cancer onset. As a result, men who develop cancer during follow-up but have not yet been diagnosed contribute to the same average PSA trajectory as cancer-free men. Their faster PSA rise is absorbed by their individual random effects rather than being attributed to an underlying biological process.*

*Gulati et al. [28] addressed this by modelling log PSA as a piecewise linear function of age, with a single slope before cancer onset and a steeper slope after onset. Adapting this idea to our joint model would let us separate two effects that are currently mixed together: how PSA changes with age in cancer-free men, and how it changes after cancer develops. This extension is challenging because cancer onset times are not directly observed and enter the model as additional random effects to be inferred.*

# Reviewer 2

Comments to the Author: The endeavour to jointly model longitudinal PSA patterns based on age and retesting is of interest. The data used is appropriate and the methodology applied is overall relevant. The manuscript is well written. I have some comments and questions that may help the authors to improve the manuscript:

**Response:** We thank the reviewer for the detailed comments. Several of the points raised concern the limits of what can be concluded from the model, particularly regarding individual-level predictions and external validation. We have sought to address these concerns throughout the manuscript. Individual responses follow below.

1. There is a substantial amount of descriptive data regarding PSA values in the methods section. This is more appropriate to present in the results section.

   **Addressed:** We agree, and Reviewer 1 raised the same point. The descriptive content has been moved to the opening of the Results section, where it now precedes Table 1. The corresponding changes are quoted in our response to Reviewer 1, comment 3.

2. How can calendar time effects during the 17 years study period influence the results? Was calendar time handled in the modelling? If not, why not?

   **Addressed:** Calendar time was not included as a covariate in the joint model. The main reason is computational: the converged fit of the model as currently specified ran for approximately one week on 20 cores using HPC resources. Adding a flexible calendar-time term to three submodels would have increased the parameter count and the runtime of an already expensive fit. A secondary consideration is that, within an individual, age and calendar time progress in parallel, and with a median of two tests per man, age and period effects are difficult to separate without strong functional-form assumptions. The age-based hazards we report should therefore be interpreted as averaged over the 2003–2020 period, and the PSA-event associations are likewise calendar-averaged.

   As a sensitivity check, we refitted both standalone time-to-event models within each of the three calendar periods used in Table 1. The hazard ratio per doubling of PSA was in the same direction and of similar magnitude in every period, indicating that the strong PSA-event associations are not driven by any single era of practice. The full details are reported in the revised Discussion and in a new section of Supporting information S5.

   We have added the sensitivity result, the methods describing it, and an explicit limitation to the Discussion [page 20, lines 357–368]:

   *"A further limitation is that calendar time was not incorporated into the model. The estimates we report should therefore be interpreted as averaged over the 2003–2020 study period. As a sensitivity check, we refitted standalone time-to-event models within each of the three calendar periods used in Table 1. The hazard ratio per doubling of PSA was in the same direction and of similar magnitude in every period: for diagnosis, 1.71 (95% CI: 1.69–1.74; $P < .001$), 1.60 (95% CI: 1.58–1.62; $P < .001$), and 1.57 (95% CI: 1.54–1.59; $P < .001$); for retesting, 1.09 (95% CI: 1.09–1.09; $P < .001$), 1.08 (95% CI: 1.08–1.08; $P < .001$), and 1.05 (95% CI: 1.05–1.05; $P < .001$), in 2003–2008, 2009–2014, and 2015–2021, respectively. The modest attenuation across periods is consistent with changes in clinical practice during the study window, including the introduction of pre-biopsy MRI and changes in opportunistic testing intensity, but a clear PSA–event association is present in every period. Full details are given in Supporting Information S5."*

3. Can differences in clinical practice within the source population create correlations between different hospitals/clinics/healthcare providers?

   **Addressed:** We expect that there is substantial heterogeneity between general practitioners and locality in PSA testing patterns. However, this level of covariation will be absorbed across the individual-level variation that is currently being modelled. This is an interesting extension to the current project that is outside of scope. We have added to the Discussion [page 19, lines 369–373]: *"We did not account for clustering by general*

*practitioner, clinic, or municipality. A multilevel analysis of PSA testing by municipality or health care provider would be an interesting extension."*

4. How was the situation handled when the interval between the available PSA-values from an individual is very short (weeks). This could likely introduce unstable PSA trajectories.

   **Addressed:** The current model does not support multiple tests on the same day, since same-day tests give a zero-length interval that is incompatible with the recurrent-event likelihood. For the few men with multiple tests on the same day, we retained the test with highest PSA value. Repeat tests within one week were rare. In our cohort, the median gap between consecutive tests is 377 days, and only 0.8% of consecutive test pairs are separated by less than one week, 4.1% by less than one month, and 11.1% by less than three months. Even when closely spaced tests do occur, we do not expect them to destabilise the fitted PSA trajectories, because the random effects modelling shrinks individual slopes toward the population mean. We have not made any changes to the manuscript.

5. What is the rationale for including the entire age range in the study population? What are the consequences of including the apparently unstable data from the extremes of the age distribution ($< 40$ and $> 90$) in the modelling exercises?

   **Addressed:** We did not apply any age-based inclusion or exclusion criteria: the cohort consists of all men with at least one PSA test in Stockholm County between 2003 and 2020. Our study aim was to describe PSA dynamics across the age range encountered in routine opportunistic testing practice. Tests at the extremes of the age distribution were uncommon: 3.66% of tests were taken in men under 40 years, and only 0.77% in men aged 90 years or older. The widening confidence band at the extremes of the age range in Figure 1 is a property of the random-effects variance function across age, not a sign that PSA values are more variable in younger men. We address this in more detail in our response to Reviewer 1, comment 2 in Results section.

6. The finding that PSA doubling time is associated with an increased likelihood for a new test is not unexpected.

   **Addressed:** We agree. This finding is consistent with clinical practice, where a rising PSA prompts more frequent retesting. We believe that the contribution of our analysis is to quantify this association while jointly accounting for the dependence between PSA, testing intensity, and diagnosis. We have now added to X: "This finding is consistent with clinical practice."

7. The expression in the results section that "uncertainty in the earlier parts of each trajectory (before the first measurement) is informed by future observations, providing a retrospective estimate of the patient's past" is a bit difficult to understand the relevance of. The authors should justify the relevance of predicting a single individual's observations, when this individual has been part of the observations used to develop the prediction model. It would likely be more relevant to display the prediction against variability of observations, and/or attempt a validation against data not used in model development, to avoid overly enthusiastic assessments of model performance.

   **Addressed:** We agree with the reviewer. This comment is closely related to comments **9** and **11**, both of which raise the same underlying concern: the original manuscript was framed too strongly toward individual predictions, while what we have done is fit a population-level joint model whose patient-level outputs are (loosely interpreted) posterior estimates of the random effects rather than validated predictions. We would like to clarify what the joint model contributes at the individual level, and why we consider this a proof of concept for future work on predictions rather than a prediction model on its own.

   A practical obstacle to using joint models of this type has been computational. Fitting them involves intractable integrals over the per-individual random effects. For a population-sized cohort with hundreds of thousands of men, this is extremely expensive. The variational approximation we use (Ormerod and Wand, 2012) replaces these intractable integrals with tractable lower-dimentional ones over per-individual variational parameters (a mean vector and covariance matrix per man) that approximate each individual's random-effects posterior. Because these variational parameters are estimated jointly with the population-level parameters during model fitting, individual-level posterior approximations are available for every man in the fitted cohort as a by-product of fitting, effectively giving us individual-level posterior estimates for free.

   What we have not done is develop a full prediction model in this study. A prediction model would require a held-out validation sample not used in model fitting, and an evaluation of calibration and discrimination, neither of which we report here. The patient-level estimates shown in the manuscript are therefore best understood as a demonstration that the variational implementation produces well-behaved individual-level posteriors at scale, and serve as a foundation for prediction-modelling work in a future study.

Accordingly, we have removed any prediction-oriented framing from the manuscript and now describe the individual-level outputs as patient-specific posterior estimates rather than predictions. The detailed changes are as follows.

- **Abstract.** We removed the claim that the joint approach yields *"more accurate ... personalised risk predictions"*, and reworded the closing sentences to refer to population-level estimates only. The revised text reads [page 3, line 42–45]: *"In conclusion, jointly modelling PSA dynamics and testing behaviour corrects for the informative observation bias inherent in opportunistic testing. This approach yields more accurate population estimates compared to traditional isolated models."*
- **Introduction.** We reworded *"we expect that predictions from the joint model will more accurately capture the underlying data-generating mechanisms"* to refer to estimates rather than predictions, and added a clarification about random effect estimates in the sentence about identifying men with faster than expected PSA rises. The revised text reads [page 5, line 77–81]: *"Specifically, we expect that estimates from the joint model will more accurately capture the underlying data-generating mechanisms than those from separate models. Patient-level posterior estimates of the random effects from such a model could serve as a basis for identifying men whose PSA is rising faster than expected for their age, signalling the need for closer clinical follow-up."*
- **Results, Fig 1 caption-line.** We reworded *"population-level predictions"* to *"population-level estimates"*, while keeping the technical term *"pointwise prediction intervals"* for the bands, since these are standard prediction intervals incorporating between-individual variance. The revised sentence reads [page 14, line 228–229]: *"The green curves show population-level estimates with 95% pointwise prediction intervals that account for both fixed-effect uncertainty and between-individual variability."*
- **Results subsection.** We renamed the subsection *"Predicted trajectories"* to [page 15, line 264] *"Patient-specific estimated trajectories"*, and reworded *"individual-specific predictions"* with [page 16, line 267]: *"individual-specific estimates"*.
- **Removed sentence.** We removed the specific sentence the reviewer flagged: *"Consequently, uncertainty in the earlier parts of each trajectory (before the first measurement) is informed by future observations, providing a retrospective estimate of the patient's past."* This sentence read as a claim about prediction, when it was describing how the random-effects posterior depends on the man's observed measurements. As the reviewer points out, this is in-sample by construction and does not support an out-of-sample interpretation. We replaced the surrounding framing with a sentence noting that the orange intervals reflect within-sample posterior estimates, not out-of-sample predictions, and pointing the reader to the Discussion for limitations on any individual-level use. The revised sentence reads [page 16, line 275–278]: *"It is important to note that these (orange) bands are within-sample posterior estimates conditional on the entire set of observed PSA values for that individual; they are not out-of-sample predictions and have not been validated as such (see Discussion for limitations on the individual-level use)."*
- **Discussion paragraphs on personalised predictions and variational approximations.** We replaced the paragraph that described the model as a tool for *"personalised decision-making"*, *"MRI referral"*, and *"dynamic risks of future testing or diagnosis"*, and merged it with the following paragraph on variational approximations. The combined revised paragraph reads [page 19, line 334–345]: *"We used variational approximations to fit the joint model. This approach scaled well to a moderately large dataset and produced patient-level posterior estimates of the random effects as a by-product of fitting. These posterior estimates summarise how an individual deviates from the population-average PSA trajectory and come with measures of uncertainty derived from the variational parameters. They are, however, within-sample, conditional on each man's full set of observed PSA values, and have not been developed or evaluated as a prediction tool in this study. They could form the basis of an individual-level prediction model in future work; such a model would additionally require a held-out validation sample not used in model fitting and an evaluation of calibration, discrimination, and clinical utility, none of which we report here. The patient-specific curves in Figs 3 and 4 should therefore be read as illustrations of the fitted random-effects structure rather than as validated predictions."*
- **Conclusions.** We have reworded the closing paragraph so it no longer claims that the joint approach *"enables individual-specific predictions of PSA evolution, retesting, and diagnostic risk"*. The revised sentence reads [page 21, line 392–394]: *"Joint modelling accounts for informative observation in opportunistic testing and yields more accurate population-level estimates of all three processes."*

Figs 3 and 4 still show two example trajectories, but we now describe them as illustrations of what the fitted random-effects structure looks like for a man without and with a prostate cancer diagnosis, rather than as predictions.

8. The figures 3 and 4 display multiple PSA values at specific ages for single individuals, and for the man diagnosed with prostate cancer, the specific age at diagnosis. This appears as information that risk being able to identify specific individuals. Please verify that such presentation of data is compatible with the ethical approval and protection of personal integrity.

   **Addressed:** Due to similar concerns, we had previously added random noise to both the ages and the PSA values. More conservatively, we have now created purely synthetic data for this plot, which are compatible with the ethical approvals and protection of personal integrity. The figure titles have been updated to read *"Estimated longitudinal and time-to-event processes from the joint model for an undiagnosed synthetic individual."* and *"Estimated longitudinal and time-to-event processes from the joint model for a diagnosed synthetic individual."*, respectively, and a corresponding sentence has been added to the main text [page 16, line 278–279] where Figs 3 and 4 are introduced: *"The individuals shown in Figs 3 and 4 are synthetic; the fitted curves were obtained from the joint model on the original data."*

9. The results presentation regarding validation of the model predictions appears limited and not in line with guidelines for reporting of prediction models. Please review relevant guidelines and preferably improve adherence to these.

   **Addressed:** We agree, and this concern is addressed in comment **7**. We have stepped back from a prediction-oriented framing of the manuscript. Full details, including the specific changes in the abstract, introduction, results, discussion, and conclusions, are given in our response to comment **7**.

10. In the discussion it is stated that "The mean age at diagnosis was 70.8 years, which explains the higher PSA densities observed among men aged 60 and older, likely reflecting cancers that had already developed by that age". There is, however, no mentioning of data on PSA density in the methods and results sections. Please clarify.

    **Addressed:** The reviewer is correct. The original text does not refer to PSA density (PSA/volume); we were thinking about probability densities (Figure S2, S3) of PSA values when writing this. We have also reworded the sentence so it is no longer phrased causally. The revised text is at [page 18, lines 309–311]:

    *"The mean age at diagnosis was 70.8 years, which is consistent with the higher PSA values observed in men aged 60 and older, likely reflecting cancers that had already developed by that age."*

11. An important concern with the current manuscript is that the results presentation and discussion allude to use of the models for individual level predictions. The validation of the models does not substantiate such claims. If the models are intended for use on the individual level a much more elaborate validation is expected, with robust support for external validity. An important restriction is how to describe and communicate the uncertainty of model predictions on the individual level. As currently presented, claims for individual level predictions have not been justified, and either the analyses should be substantially expanded or these claims be removed. The statement that "This approach scaled well to a moderately large dataset and supported personalised predictions, including measures of uncertainty" is not agreed.

    **Addressed:** We agree, and this comment is closely linked to comments **7** and **9**. We have removed the claims for individual-level predictions throughout the manuscript, including the specific sentence the reviewer quotes: *"This approach scaled well to a moderately large dataset and supported personalised predictions, including measures of uncertainty"* has been replaced with text describing the variational fit as producing patient-level posterior estimates of the random effects with corresponding measures of uncertainty, while explicitly noting that these have not been developed or evaluated as a prediction tool. Full details of the textual changes, and our reasoning, are given in our response to comment **7**.

# Other changes

Apart from comments from the reviewers, in the course of preparing this revision we found the following errors in the manuscript:

- **Misspelled author name** [page 1, line 5]: we have corrected the spelling.

- **Table 1**: [page 13, line 224] the "Biopsies total" and "Diagnosed total" columns, and the corresponding per-PY rates, were inflated because the underlying summary counted PSA test rows rather than distinct men per cell. We have corrected the table so that these columns now count each man once per (period, age group) cell. The PSA, person-years, and testing columns were not affected.

# References


Gulati, R., Inoue, L., Katcher, J., Hazelton, W., and Etzioni, R. (2010). Calibrating disease progression models using population data: a critical precursor to policy development in cancer control. *Biostatistics*, 11(4):707–719.

Ormerod, J. T. and Wand, M. P. (2012). Gaussian variational approximate inference for generalized linear mixed models. *Journal of Computational and Graphical Statistics*, 21(1):2–17.
