# Supplementary material for: Joint modelling of PSA dynamics and prostate cancer risks: A population-based study in men without prior prostate cancer
Source: PLoS One. 2026 Jul 10;21(7):e0343751. doi: 10.1371/journal.pone.0343751 (PMC13354087; doi:10.1371/journal.pone.0343751)
Supplement: Peer review history

# Peer review: Decision Letter 0

**DOI:** 10.1371/journal.pone.0343751.r001

**Authors:** Stanisław Wroński

14 May 2026

PONE-D-26-05935

Joint modelling of PSA dynamics and prostate cancer risks: A population-based study

PLOS One

Dear Dr. Birzhan Akynkozhayev,

Thank you for submitting your manuscript to PLOS ONE. After careful consideration, we feel that it has merit but does not fully meet PLOS ONE’s publication criteria as it currently stands. Therefore, we invite you to submit a revised version of the manuscript that addresses the points raised during the review process.

Please submit your revised manuscript by 15th June 2026. If you will need more time than this to complete your revisions, please reply to this message or contact the journal office at plosone@plos.org. When you're ready to submit your revision, log on to https://www.editorialmanager.com/pone/ and select the 'Submissions Needing Revision' folder to locate your manuscript file.

Please include the following items when submitting your revised manuscript:

- A letter that responds to each point raised by the academic editor and reviewer(s). You should upload this letter as a separate file labeled 'Response to Reviewers'.
- A marked-up copy of your manuscript that highlights changes made to the original version. You should upload this as a separate file labeled 'Revised Manuscript with Track Changes'.
- An unmarked version of your revised paper without tracked changes. You should upload this as a separate file labeled 'Manuscript'.

If you would like to make changes to your financial disclosure, please include your updated statement in your cover letter. Guidelines for resubmitting your figure files are available below the reviewer comments at the end of this letter.

If applicable, we recommend that you deposit your laboratory protocols in protocols.io to enhance the reproducibility of your results. Protocols.io assigns your protocol its own identifier (DOI) so that it can be cited independently in the future. For instructions see: https://journals.plos.org/plosone/s/submission-guidelines#loc-laboratory-protocols. Additionally, PLOS ONE offers an option for publishing peer-reviewed Lab Protocol articles, which describe protocols hosted on protocols.io. Read more information on sharing protocols at https://plos.org/protocols?utm_medium=editorial-email&utm_source=authorletters&utm_campaign=protocols.

As the corresponding author, your ORCID iD is verified in the submission system and will appear in the published article. PLOS supports the use of ORCID, and we encourage all coauthors to register for an ORCID iD and use it as well. Please encourage your coauthors to verify their ORCID iD within the submission system before final acceptance, as unverified ORCID iDs will not appear in the published article. *Only*  the individual author can complete the verification step; PLOS staff *cannot*  verify ORCID iDs on behalf of authors.

We look forward to receiving your revised manuscript.

Kind regards,

Stanisław Jacek Wroński, M.D., Ph.D, FEBU

Academic Editor

PLOS One

Journal Requirements:

When submitting your revision, we need you to address these additional requirements.

1. Please ensure that your manuscript meets PLOS ONE's style requirements, including those for file naming. The PLOS ONE style templates can be found at

https://journals.plos.org/plosone/s/file?id=wjVg/PLOSOne_formatting_sample_main_body.pdf and

https://journals.plos.org/plosone/s/file?id=ba62/PLOSOne_formatting_sample_title_authors_affiliations.pdf

2. Please provide additional details regarding participant consent. In the ethics statement in the Methods and online submission information, please ensure that you have specified (1) whether consent was informed and (2) what type you obtained (for instance, written or verbal, and if verbal, how it was documented and witnessed). If your study included minors, state whether you obtained consent from parents or guardians. If the need for consent was waived by the ethics committee, please include this information.

If you are reporting a retrospective study of medical records or archived samples, please ensure that you have discussed whether all data were fully anonymized before you accessed them and/or whether the IRB or ethics committee waived the requirement for informed consent. If patients provided informed written consent to have data from their medical records used in research, please include this information.

3. Thank you for stating the following financial disclosure:

"Holly Edward is a recipient of the Canadian Institutes of Health Research’s Doctoral Research Award (Funding Reference Number: 193451)."

Please state what role the funders took in the study.  If the funders had no role, please state: "The funders had no role in study design, data collection and analysis, decision to publish, or preparation of the manuscript."

If this statement is not correct you must amend it as needed.

Please include this amended Role of Funder statement in your cover letter; we will change the online submission form on your behalf.

4. In this instance it seems there may be acceptable restrictions in place that prevent the public sharing of your minimal data. However, in line with our goal of ensuring long-term data availability to all interested researchers, PLOS’ Data Policy states that authors cannot be the sole named individuals responsible for ensuring data access (http://journals.plos.org/plosone/s/data-availability#loc-acceptable-data-sharing-methods).

Data requests to a non-author institutional point of contact, such as a data access or ethics committee, helps guarantee long term stability and availability of data. Providing interested researchers with a durable point of contact ensures data will be accessible even if an author changes email addresses, institutions, or becomes unavailable to answer requests.

Before we proceed with your manuscript, please also provide non-author contact information (phone/email/hyperlink) for a data access committee, ethics committee, or other institutional body to which data requests may be sent. If no institutional body is available to respond to requests for your minimal data, please consider if there any institutional representatives who did not collaborate in the study, and are not listed as authors on the manuscript, who would be able to hold the data and respond to external requests for data access? If so, please provide their contact information (i.e., email address). Please also provide details on how you will ensure persistent or long-term data storage and availability.

5. Thank you for stating the following in the Competing Interests section:

"I have read the journal's policy and the authors of this manuscript have the following competing interests: Dr Nordström reported owning shares in A3P Biomedical. No other disclosures were reported. This does not alter our adherence to PLOS ONE policies on sharing data and materials."

Please confirm that this does not alter your adherence to all PLOS ONE policies on sharing data and materials, by including the following statement: "This does not alter our adherence to  PLOS ONE policies on sharing data and materials.” (as detailed online in our guide for authors http://journals.plos.org/plosone/s/competing-interests).  If there are restrictions on sharing of data and/or materials, please state these. Please note that we cannot proceed with consideration of your article until this information has been declared.

Please include your updated Competing Interests statement in your cover letter; we will change the online submission form on your behalf.

6. We note that you have indicated that there are restrictions to data sharing for this study. PLOS only allows data to be available upon request if there are legal or ethical restrictions on sharing data publicly. For more information on unacceptable data access restrictions, please see http://journals.plos.org/plosone/s/data-availability#loc-unacceptable-data-access-restrictions.

Before we proceed with your manuscript, please address the following prompts:

a) If there are ethical or legal restrictions on sharing a de-identified data set, please explain them in detail (e.g., data contain potentially identifying or sensitive patient information, data are owned by a third-party organization, etc.) and who has imposed them (e.g., a Research Ethics Committee or Institutional Review Board, etc.). Please also provide contact information for a data access committee, ethics committee, or other institutional body to which data requests may be sent.

b) If there are no restrictions, please upload the minimal anonymized data set necessary to replicate your study findings to a stable, public repository and provide us with the relevant URLs, DOIs, or accession numbers. For a list of recommended repositories, please see

https://journals.plos.org/plosone/s/recommended-repositories. You also have the option of uploading the data as Supporting Information files, but we would recommend depositing data directly to a data repository if possible.

We will update your Data Availability statement on your behalf to reflect the information you provide.

7. If the reviewer comments include a recommendation to cite specific previously published works, please review and evaluate these publications to determine whether they are relevant and should be cited. There is no requirement to cite these works unless the editor has indicated otherwise.

Reviewers' comments:

Reviewer's Responses to Questions

**Comments to the Author**

1. Is the manuscript technically sound, and do the data support the conclusions?

The manuscript must describe a technically sound piece of scientific research with data that supports the conclusions. Experiments must have been conducted rigorously, with appropriate controls, replication, and sample sizes. The conclusions must be drawn appropriately based on the data presented.

Reviewer #1: Yes

Reviewer #2: Partly

**********

2. Has the statistical analysis been performed appropriately and rigorously?

Reviewer #1: Yes

Reviewer #2: Yes

**********

3. Have the authors made all data underlying the findings in their manuscript fully available?

The PLOS Data policy requires authors to make all data underlying the findings described in their manuscript fully available without restriction, with rare exception (please refer to the Data Availability Statement in the manuscript PDF file). The data should be provided as part of the manuscript or its supporting information, or deposited to a public repository. For example, in addition to summary statistics, the data points behind means, medians and variance measures should be available. If there are restrictions on publicly sharing data—e.g. participant privacy or use of data from a third party—those must be specified.

Reviewer #1: Yes

Reviewer #2: No

**********

4. Is the manuscript presented in an intelligible fashion and written in standard English?

PLOS ONE does not copyedit accepted manuscripts, so the language in submitted articles must be clear, correct, and unambiguous. Any typographical or grammatical errors should be corrected at revision, so please note any specific errors here.

Reviewer #1: Yes

Reviewer #2: Yes

**********

5. Review Comments to the Author

Please use the space provided to explain your answers to the questions above. You may also include additional comments for the author, including concerns about dual publication, research ethics, or publication ethics. (Please upload your review as an attachment if it exceeds 20,000 characters)

Reviewer #1: The authors have analyzed PSA testing dynamics by applying a joint model of PSA values and testing patterns and risk of prostate cancer to observational data. The model identified stronger associations between PSA increase and prostate cancer diagnosis and retesting. They concluded that the joint approach is more appropriate compared to separate models and that PSA dynamics may be clinically informative.

The manuscript is well written and brings an important contribution to the field of modelling of PSA dynamics and screening. I have some (mainly minor) comments that may improve the manuscript.

Title

I suggest that you add the target population to the title – for example:

“Joint modelling of PSA dynamics and prostate cancer risks: A population-based study of men without prior prostate cancer diagnosis”

Abstract

Without having read the rest of the manuscript, it is not entirely clear how the conclusions are supported by the results presented in the abstract alone. How does the joint model correct for bias, and in what sense is it more accurate? Could this be demonstrated with results, for example by explicitly comparing it with the separate models?

Minor: I do not agree that the following is a limitation “the model does not explicitly account for PSA trajectory changes after cancer onset” considering that you aimed to model PSA dynamics in men without prior prostate cancer diagnosis. It could perhaps be mentioned as a delimitation in the discussion.

Introduction

Minor: The last paragraph – I suggest that you add the target population to the aim, for example something like this:

“In this study, we aimed to develop a detailed population-based PSA model for men without a prior prostate cancer diagnosis.”

Materials and methods

The authors state that a PSA is followed by an MRI and then possibly by a biopsy. At the same time data on men that underwent PSA testing was collected between 2003-2020. MRI was only introduced into the diagnostic pathway during the latter part of the study window. I suggest that the authors clarify how the guidelines have changed over the study period and provide details on when MRI was introduced.

Minor: Also, not all men should undergo an MRI before biopsy, so I suggest that you say that the contemporary typical diagnostic pathway is PSA – MRI – biopsy.

Minor: I would say that the paragraphs between lines 97 and 113 are results and should be moved to the results section.

Minor: what was the lower detection limit for PSA from the laboratories and did this vary over calendar time? Was there an upper reported limit for PSA? How did you handle this in the modelling?

Given the emphasis on the novelty of the modelling approach, I would have appreciated a bit more detail about the model in the main text under “Statistical methods”. For instance, how were the spline knots chosen, and how was the log transformed PSA trajectory explicitly incorporated into the subsequent Cox models?

Minor: It would also have been good with a sentence or two about how the model was fitted, and a short description of how you plan to illustrate or present the fitted model(s). This would be valuable especially to those unfamiliar with the joint/latent modelling approach.

Why did the separate models not include splines, or did they? It is not clear based on reading lines 135-139. I would have expected the separate models to be as similar as possible to the joint model in terms of how the covariates were represented.

Minor: what was the covariance matrix for the random effects in the longitudinal submodel?

Minor: supplement – I think there should be an explicit reference to the arXiv article that describes the method with the relevant identifier.

Results

The results are clearly presented. However, I believe it would be valuable to include an explicit comparison between the joint model and the separate models in the manuscript, especially since this is a key finding highlighted in both the abstract and the discussion’s summary of results. Perhaps Figures 1 and 3–4 could be combined to make space for additional details on all the models currently found in the supplement.

Minor: how come there is such a wide interval for PSA for younger ages in Figure 1? Is it for the same reason as for the later figures, or is there a larger heterogeneity in younger men?

Minor: did model performance somehow deteriorate or improve over calendar time, considering the changing diagnostic landscape and in particular the introduction of MRI?

Discussion/conclusions

Minor: consider to give an example of how the model can be used in a personalized decision-making context?

I would have appreciated a more in‑depth discussion of the modelling choices and the plausibility of the underlying assumptions. For example, the authors conclude the discussion by stating that they plan to model cancer onset and integrate it into the joint model. It would be helpful for readers to understand why this addition would be valuable—for example, in what ways might it improve model performance? Could the authors illustrate how the current model may underperform, perhaps by showing that it does not fully capture the heterogeneity and dynamics in the data over time, and how the proposed extension might address this limitation?

Reviewer #2: The endeavour to jointly model longitudinal PSA patterns based on age and retesting is of interest. The data used is appropriate and the methodology applied is overall relevant. The manuscript is well written. I have some comments and questions that may help the authors to improve the manuscript:

1. There is a substantial amount of descriptive data regarding PSA values in the methods section. This is more appropriate to present in the results section.

2. How can calendar time effects during the 17 years study period influence the results? Was calendar time handled in the modelling? If not, why not?

3. Can differences in clinical practice within the source population create correlations between different hospitals/clinics/healthcare providers?

4. How was the situation handled when the interval between the available PSA-values from an individual is very short (weeks). This could likely introduce unstable PSA trajectories.

5. What is the rationale for including the entire age range in the study population? What are the consequences of including the apparently unstable data form the extremes of the age distribution (<40 and >90) in the modelling exercises?

6. The finding that PSA doubling time is associated with an increased likelihood for a new test is not unexpected.

7. The expression in the results section that “uncertainty in the earlier parts of each trajectory (before the first measurement) is informed by future observations, providing a retrospective estimate of the patient’s past” is a bit difficult to understand the relevance of. The authors should justify the relevance of predicting a single individual’s observations, when this individual has been part of the observations used to develop the prediction model. It would likely be more relevant to display the prediction against variability of observations, and/or attempt a validation against data not used in model development, to avoid overly enthusiastic assessments of model performance.

8. The figures 3 and 4 display multiple PSA values at specific ages for single individuals, and for the man diagnosed with prostate cancer, the specific age at diagnosis. This appears as information that risk being able to identify specific individuals. Please verify that such presentation of data is compatible with the ethical approval and protection of personal integrity.

9. The results presentation regarding validation of the model predictions appears limited and not in line with guidelines for reporting of prediction models. Please review relevant guidelines and preferably improve adherence to these.

10. In the discussion it is stated that “The mean age at diagnosis was 70.8 years, which explains the higher PSA densities observed among men aged 60 and older, likely reflecting cancers that had already developed by that age”. There is, however, no mentioning of data on PSA density in the methods and results sections. Please clarify.

11. An important concern with the current manuscript is that the results presentation and discussion allude to use of the models for individual level predictions. The validation of the models does not substantiate such claims. If the models are intended for use on the individual level a much more elaborate validation is expected, with robust support for external validity. An important restriction is how to describe and communicate the uncertainty of model predictions on the individual level. As currently presented, claims for individual level predictions have not been justified, and either the analyses should be substantially expanded or these claims be removed. The statement that “This approach scaled well to a moderately large dataset and supported personalised predictions, including measures of uncertainty” is not agreed.

**********

6. PLOS authors have the option to publish the peer review history of their article (what does this mean?). If published, this will include your full peer review and any attached files.

If you choose “no”, your identity will remain anonymous but your review may still be made public.

**Do you want your identity to be public for this peer review?**  For information about this choice, including consent withdrawal, please see our Privacy Policy.

Reviewer #1: No

Reviewer #2: No

**********

[NOTE: If reviewer comments were submitted as an attachment file, they will be attached to this email and accessible via the submission site. Please log into your account, locate the manuscript record, and check for the action link "View Attachments". If this link does not appear, there are no attachment files.]

To ensure your figures meet our technical requirements, please review our figure guidelines: https://journals.plos.org/plosone/s/figures

You may also use PLOS’s free figure tool, NAAS, to help you prepare publication quality figures: https://journals.plos.org/plosone/s/figures#loc-tools-for-figure-preparation.

NAAS will assess whether your figures meet our technical requirements by comparing each figure against our figure specifications.

# Author comment: Author response to Decision Letter 1

**DOI:** 10.1371/journal.pone.0343751.r002

27 May 2026

Dear Editor,

Thank you for the opportunity to submit our revised manuscript.

As our detailed point-by-point responses to the reviewers' comments contain mathematical formulae and specific formatting that cannot be rendered correctly within this plain text box, we have provided our complete response in the attached "Response_to_Reviewers.pdf" document.

Please also refer to the attached "Cover_Letter_Revision.docx" for our responses to the journal-level requirements.

Thank you for your time and consideration.

Attachment Submitted filename: Response_to_Reviewers.pdf

# Peer review: Decision Letter 1

**DOI:** 10.1371/journal.pone.0343751.r003

**Authors:** Stanisław Wroński, Stanisław Wroński

29 Jun 2026

Joint modelling of PSA dynamics and prostate cancer risks: A population-based study in men without prior prostate cancer

PONE-D-26-05935R1

Dear Dr. B. Akynkozhayev

We’re pleased to inform you that your manuscript has been judged scientifically suitable for publication and will be formally accepted for publication once it meets all outstanding technical requirements.

Within one week, you’ll receive an e-mail detailing the required amendments. When these have been addressed, you’ll receive a formal acceptance letter and your manuscript will be scheduled for publication.

An invoice will be generated when your article is formally accepted. Please note, if your institution has a publishing partnership with PLOS and your article meets the relevant criteria, all or part of your publication costs will be covered. Please make sure your user information is up-to-date by logging into Editorial Manager at Editorial Manager® and clicking the ‘Update My Information' link at the top of the page. For questions related to billing, please contact billing support.

If your institution or institutions have a press office, please notify them about your upcoming paper to help maximize its impact. If they’ll be preparing press materials, please inform our press team as soon as possible -- no later than 48 hours after receiving the formal acceptance. Your manuscript will remain under strict press embargo until 2 pm Eastern Time on the date of publication. For more information, please contact onepress@plos.org.

Kind regards,

Stanisław Jacek Wroński, M.D., Ph.D, FEBU

Academic Editor

PLOS One

Reviewers' comments:

Reviewer's Responses to Questions

**Comments to the Author**

1. If the authors have adequately addressed your comments raised in a previous round of review and you feel that this manuscript is now acceptable for publication, you may indicate that here to bypass the “Comments to the Author” section, enter your conflict of interest statement in the “Confidential to Editor” section, and submit your "Accept" recommendation.

Reviewer #2: All comments have been addressed

**********

2. Is the manuscript technically sound, and do the data support the conclusions?

The manuscript must describe a technically sound piece of scientific research with data that supports the conclusions. Experiments must have been conducted rigorously, with appropriate controls, replication, and sample sizes. The conclusions must be drawn appropriately based on the data presented.

Reviewer #2: (No Response)

**********

3. Has the statistical analysis been performed appropriately and rigorously?

Reviewer #2: (No Response)

**********

4. Have the authors made all data underlying the findings in their manuscript fully available?

The PLOS Data policy requires authors to make all data underlying the findings described in their manuscript fully available without restriction, with rare exception (please refer to the Data Availability Statement in the manuscript PDF file). The data should be provided as part of the manuscript or its supporting information, or deposited to a public repository. For example, in addition to summary statistics, the data points behind means, medians and variance measures should be available. If there are restrictions on publicly sharing data—e.g. participant privacy or use of data from a third party—those must be specified.

Reviewer #2: (No Response)

**********

5. Is the manuscript presented in an intelligible fashion and written in standard English?

PLOS ONE does not copyedit accepted manuscripts, so the language in submitted articles must be clear, correct, and unambiguous. Any typographical or grammatical errors should be corrected at revision, so please note any specific errors here.

Reviewer #2: (No Response)

**********

6. Review Comments to the Author

Please use the space provided to explain your answers to the questions above. You may also include additional comments for the author, including concerns about dual publication, research ethics, or publication ethics. (Please upload your review as an attachment if it exceeds 20,000 characters)

Reviewer #2: (No Response)

**********

7. PLOS authors have the option to publish the peer review history of their article (what does this mean?). If published, this will include your full peer review and any attached files.

If you choose “no”, your identity will remain anonymous but your review may still be made public.

**Do you want your identity to be public for this peer review?**  For information about this choice, including consent withdrawal, please see our Privacy Policy.

Reviewer #2: **Yes:** Rolf Gedeborg

**********

# Editor report: Acceptance letter

**DOI:** 10.1371/journal.pone.0343751.r004

**Authors:** Stanisław Wroński, Stanisław Wroński

PONE-D-26-05935R1

PLOS One

Dear Dr. Akynkozhayev,

I'm pleased to inform you that your manuscript has been deemed suitable for publication in PLOS One. Congratulations! Your manuscript is now being handed over to our production team.

At this stage, our production department will prepare your paper for publication. This includes ensuring the following:

* All references, tables, and figures are properly cited

* All relevant supporting information is included in the manuscript submission,

* There are no issues that prevent the paper from being properly typeset

You will receive further instructions from the production team, including instructions on how to review your proof when it is ready. Please keep in mind that we are working through a large volume of accepted articles, so please give us a few days to review your paper and let you know the next and final steps.

Lastly, if your institution or institutions have a press office, please let them know about your upcoming paper now to help maximize its impact. If they'll be preparing press materials, please inform our press team within the next 48 hours. Your manuscript will remain under strict press embargo until 2 pm Eastern Time on the date of publication. For more information, please contact onepress@plos.org.

You will receive an invoice from PLOS for your publication fee after your manuscript has reached the completed accept phase. If you receive an email requesting payment before acceptance or for any other service, this may be a phishing scheme. Learn how to identify phishing emails and protect your accounts at https://explore.plos.org/phishing.

If we can help with anything else, please email us at customercare@plos.org.

Thank you for submitting your work to PLOS ONE and supporting open access.

Kind regards,

PLOS ONE Editorial Office Staff

on behalf of

Dr. Stanisław Jacek Wroński

Academic Editor

PLOS One
